# Rapid factor depletion highlights intricacies of nucleoplasmic RNA degradation

**DOI:** 10.1093/nar/gkac001

**Published:** 2022-01-20

**Authors:** Maria Gockert, Manfred Schmid, Lis Jakobsen, Marvin Jens, Jens S Andersen, Torben Heick Jensen

**Affiliations:** Department of Molecular Biology and Genetics, Aarhus University, C.F. Møllers Allé 3, Building 1130, 8000 Aarhus C, Denmark; Department of Molecular Biology and Genetics, Aarhus University, C.F. Møllers Allé 3, Building 1130, 8000 Aarhus C, Denmark; Department of Biochemistry and Molecular Biology, University of Southern Denmark, Campusvej 55, 5230 Odense M, Denmark; Department of Biology, Massachusetts Institute of Technology, 31 Ames Street, 68-271A, Cambridge, MA 02139-4307, USA; Department of Biochemistry and Molecular Biology, University of Southern Denmark, Campusvej 55, 5230 Odense M, Denmark; Department of Molecular Biology and Genetics, Aarhus University, C.F. Møllers Allé 3, Building 1130, 8000 Aarhus C, Denmark

## Abstract

Turnover of nucleoplasmic transcripts by the mammalian multi-subunit RNA exosome is mediated by two adaptors: the Nuclear EXosome Targeting (NEXT) complex and the Poly(A) tail eXosome Targeting (PAXT) connection. Functional analyses of NEXT and PAXT have largely utilized long-term factor depletion strategies, facilitating the appearance of indirect phenotypes. Here, we rapidly deplete NEXT, PAXT and core exosome components, uncovering the direct consequences of their acute losses. Generally, proteome changes are sparse and largely dominated by co-depletion of other exosome and adaptor subunits, reflecting possible subcomplex compositions. While parallel high-resolution 3′ end sequencing of newly synthesized RNA confirms previously established factor specificities, it concomitantly demonstrates an inflation of long-term depletion datasets by secondary effects. Most strikingly, a general intron degradation phenotype, observed in long-term NEXT depletion samples, is undetectable upon short-term depletion, which instead emphasizes NEXT targeting of snoRNA-hosting introns. Further analysis of these introns uncovers an unusual mode of core exosome-independent RNA decay. Our study highlights the accumulation of RNAs as an indirect result of long-term decay factor depletion, which we speculate is, at least partly, due to the exhaustion of alternative RNA decay pathways.

## INTRODUCTION

The pervasive nature of RNA polymerase II (RNAPII) transcription generates a cornucopia of diverse transcripts (1,2). To maintain cellular homeostasis, this vast transcriptional output has to be dampened by RNA degradation (3–6). In the nucleus, such decay is predominantly conducted by the highly conserved RNA exosome, a 3′–5′ exo- and endo-ribonucleolytic multi-subunit complex, that targets export-restrained protein coding RNA as well as a large ensemble of long noncoding RNA (lncRNA). In addition, the nuclear exosome partakes in the maturation of tRNA, rRNA and sn(o)RNA (3,7–9). In mammalian cells, exosome catalytic activity is provided by the DIS3 and EXOSC10 nucleases located on either end of the barrel-shaped core complex (10–12). The processive nuclease DIS3 is tethered to the exit channel of the exosome where it is exposed to RNA threaded through the exosome core (13,14). The distributive EXOSC10 exonuclease is located at the exosome ‘entrance’ where it can access substrates without core threading, although its physical presence may still be relevant for DIS3 activity (15). The predominantly nucleoplasmic DIS3 (16) is essential for most decay activities of the nuclear exosome, while the nucleolar-enriched EXOSC10 primarily targets short 3′ extended RNA precursors and appears to contribute to lncRNA degradation only after long-term impairment of DIS3 (10). Attesting to their physiological importance, mutations of various exosome subunits and exosome cofactors have been tightly linked to human disease (17).

To be able to engage with its many and diverse substrates, the exosome associates with compartment-specific adaptors. In the nucleoplasm, two such adaptors have been identified: the Nuclear EXosome Targeting (NEXT) complex and the Poly(A) tail eXosome Targeting (PAXT) connection (18,19). A central component of both of these adaptors is the RNA helicase MTR4 (MTREX, SKIV2L2), which contacts the RNA exosome and unwinds substrates prior to their exosomal degradation (20,21). The stable trimeric NEXT complex is further composed of the zinc finger protein ZCCHC8 and the small RNA binding protein RBM7 (18). In contrast to this well-defined composition of NEXT, the PAXT connection displays a more subtle architecture. A core complex appears to consist of a tightly bound heterodimer of MTR4 and the zinc finger protein ZFC3H1, which is then complemented by more transient associations with various RNA binding proteins, including the zinc finger protein ZC3H3, one of the two paralogous RNA binding proteins, RBM26 or RBM27, and an RNA-dependent interaction with the nuclear poly(A) binding protein, PABPN1 (19,22). Both NEXT and PAXT can further be linked to the ARS2-bound nuclear cap-binding complex (CBCA), also engaging the ZC3H18 protein (19,23), and thus efficiently promoting degradation of short capped transcripts, such as promotor upstream transcripts (PROMPTs), enhancer RNAs (eRNAs) and otherwise prematurely terminated (pt) transcripts (18,19,24,25).

NEXT- and PAXT-sensitive RNAs have been thoroughly characterized (19,22,25–28). Generally, the NEXT complex targets RNAs with unprotected and nonadenylated (pA^−^) 3′ ends, exemplified by transcripts produced by the aforementioned PROMPT and eRNA loci as well as the large amount of TSS-proximal transcription termination at RNAPII transcription units (TUs) (29). Furthermore, the biogenesis of snoRNAs generates a sizable number of pA^−^ and NEXT-sensitive processing intermediates (24). This is because the majority of mammalian snoRNAs are hosted within introns of long RNAPII transcripts and their maturation relies on the splicing, debranching and exonucleolytic trimming of intronic 5′- and 3′-flanking sequences (30,31). While information about 5′–3′ trimming mechanisms is limited (30,32,33), some lines of evidence have implicated the RNA exosome and NEXT in the 3′–5′ exonucleolysis of snoRNA-hosting introns, and possibly also regular introns (24,34,35). In contrast to this vast production of pA^−^ ends, polyadenylated (pA^+^) RNA 3′ ends mostly originate from cleavage and polyadenylation-dependent processing (29,36). If the resulting transcript is not rapidly exported to the cytoplasm, such pA^+^ 3′ ends can be subject to PAXT-mediated degradation, which targets many lncRNAs, PROMPTs, eRNAs, ptRNAs and a subset of mRNAs (19,22,25–27,37). Finally, even though their molecular recognition is distinct, NEXT and PAXT substrates are not strictly separated transcript classes, and resultingly, NEXT- and PAXT-sensitive isoforms are frequently and promiscuously produced from within the same TUs (26). Moreover, the two exosome adaptors can act cooperatively, when pA^−^ transcripts, escaping NEXT activity, undergo post-transcriptional polyadenylation and enter a ‘fail-safe’ PAXT-dependent pathway (26).

The majority of published loss-of-function studies to interrogate nucleoplasmic RNA degradation in human cells have relied on RNA interference (RNAi) depletions, requiring extended time periods of up to several days. Recently, more rapid depletion approaches have been developed that can directly target a protein for degradation within minutes to hours (38,39). This allows for the examination of acute and direct consequences of the loss-of-function phenotype, circumventing interference from gradually accumulating indirect effects or any phenotypic masking through compensation that can arise over longer timescales. One such rapid depletion method relies on the auxin-inducible degron (AID) system, where the specific depletion of an AID-fused protein of interest is induced in the presence of auxin/indole-3-acetic acid (IAA) (40). Protein decay is then triggered by the AID effector protein TIR1, a member of the SCF^TIR1^ E3 ligase complex, which specifically recognizes IAA-bound AID tags and triggers ubiquitin-mediated proteasomal degradation of the AID-tagged protein (40,41).

Here, we compare global proteomic and transcriptomic phenotypes following AID- or RNAi-mediated depletions of ZCCHC8 (NEXT), ZFC3H1 (PAXT) or EXOSC3, a core component of the RNA exosome. Our proteomic analysis reveals co-depletion of factors intimately engaged with the depleted decay component in question, but few other short-term changes. At the transcriptomic level, rapid depletion of ZCCHC8 or ZFC3H1 emphasizes the interplay between decay pathways and exposes/highlights substrate handover from NEXT to PAXT as an immediate consequence of ZCCHC8 depletion. By intersecting RNAi and AID depletion data, we further distinguish targets that are only identified upon long-term factor depletion, suggesting that their upregulation may be indirect. These targets include 3′ ends of short introns, which are, in contrast to their long or snoRNA-hosting counterparts, primarily detected in RNAi experiments. Focused analysis of 3′ ends at snoRNA-hosting introns uncovers nonprocessive trimming, which occurs in the absence of the core exosome component EXOSC3. We provide evidence that EXOSC10 and the poly(A)-specific ribonuclease (PARN) can partake in this 3′ end trimming. Overall, our data reveal the accumulation of indirect substrates during long-term RNA decay factor depletion, highlighting the need for short-term depletion strategies to yield a more precise insight into global transcriptomic phenotypes.

## MATERIALS AND METHODS

### Cell cultures and lines

HeLa cell lines expressing TIR1 (HeLa:TIR1) were kindly provided by Prof. Edouard Bertrand. Cells were maintained in Dulbecco’s modified Eagle’s medium supplemented with 10% fetal bovine serum (FBS) and 1% penicillin/streptomycin (P/S) at 37°C and 5% CO_2_. For CRISPR/Cas9 manipulation, 5 × 10^5^ cells were seeded per well in a six-well plate in dimethyl sulfoxide + FBS and transfected with 1 μg of each repair plasmid (see below) and 1 μg CRISPR/Cas9 plasmid using Lipofectamine^®^ 2000 or Lipofectamine^®^ 3000 reagent (Invitrogen/Thermo Fisher). For CRISPR/Cas9 knock-in (KI) experiments, two repair templates, carrying different selection markers (hygromycin and neomycin resistance), were co-transfected to ensure tagging of at least two alleles upon double antibiotic selection, thereby increasing KI efficiency. Cas9 plasmid was px330 and included respective sgRNA sequence [see Supplementary Table S1, with assembly as described (42)]. Twenty-four hours after lipid transfection, the medium was replaced with medium containing 1% P/S. Two days after lipid transfection, the cells were subjected to antibiotic selection with 100 μg/ml hygromycin (Invitrogen/Thermo Fisher) and 700 μg/ml G418 (Gibco/Thermo Fisher). Upon complete selection, single colonies were isolated by limiting dilution and later screened by genotyping PCR (for primers, see Supplementary Table S2) using Phusion^®^ Polymerase (NEB) following the manufacturer’s instructions.

For AID-mediated protein depletion, 750 μM IAA sodium salt (Sigma-Aldrich) was added to the culture medium for the indicated time periods. For RNAi, an initial transfection of 20 nM siRNA using siLentFectTM (Bio-Rad) was followed 2 days later by a second siRNA transfection using Lipofectamine^®^ 2000 (Invitrogen/Thermo Fisher). Cells were treated for a total of 4 days, and for both transfections, the respective manufacturer’s guidelines were followed. All siRNA sequences are listed in Supplementary Table S3.

### Repair template design and assembly

The repair plasmids were assembled as follows: SalI restriction site (RS), homology arm, GGSG linker, KpnI RS, mAID, GSG linker, T2A, NheI RS, hygromycin or neomycin resistance marker, XhoI RS homology arm and MluI RS. The mAID tag was templated within the original full-length degron according to (43) from a plasmid (pMK38) kindly provided by Prof. Masato Kanemaki. The T2A-NheI-Neo residence marker region was templated from a plasmid kindly provided by Prof. David Bentley. All additional features were introduced through respective design of PCR oligos. Templates for PCR, plasmid DNA or genomic DNA (gDNA) were purified using the GeneJET Plasmid Miniprep Kit (Thermo Fisher) or the PureLink^®^ DNA Mini Kit (Invitrogen), respectively. For all PCR reactions, Phusion^®^ High-Fidelity Polymerase (NEB) was used following the manufacturer’s instructions. Initially, a ‘scaffold’ vector was constructed using NEBuilder HiFi DNA Assembly (NEB), including restriction digest of the backbone and PCR-generated inserts with 20-nt homology overhangs on both sides. All subsequent target-specific repair templates were generated by switching of the homology arms or resistance cassettes by conventional cloning. Correct assembly was confirmed by Sanger sequencing.

### Cell lysis, protein aggregation capture and digestion

Frozen cell pellets were thawed and lysed in warm lysis buffer [5% (w/v) sodium dodecyl sulfate, 100 mM Tris hydrochloride, pH 8.5, 5 mM tris(2-carboxyethyl)phosphine, 10 mM 2-chloroacetamide]. The cell lysate was heated at 99°C for 10 min followed by microtip probe sonication (Qsonica) for 5 s at 20% amplitude. Protein digestion was performed by automated protein aggregation capture digestion on a KingFisher Duo Prime robot (Thermo Scientific) (44). Samples were diluted with water and added to acetonitrile to a final concentration of 70% as well as MagResyn Hydroxyl beads. Protein aggregation was performed in two loops with 1 min mixing at medium speed followed by 10 min pause. The beads were subsequently washed three times in 95% acetonitrile and two times in 70% ethanol without releasing the beads from the magnets. Each washing step consisted of 2.5 min mixing at slow speed (45). The beads were released into 50 mM ammonium bicarbonate with LysC (Wako) and trypsin (Promega) and digestion was carried out at room temperature overnight. Samples were centrifuged and peptide concentrations were measured/estimated using a NanoPhotometer N60 (Implen).

### Isobaric peptide labeling for relative quantification (TMTpro 16-plex)

Peptides were chemically labeled by on-column TMT labeling (46–48). StageTips were packed with C18 disks (3M), which were conditioned with methanol followed by 60% acetonitrile, 0.5% acetic acid and subsequently equilibrated with 1% trifluoroacetic acid. Samples were diluted in 1% trifluoroacetic acid and 15 μg was loaded on StageTip columns by centrifugation. The bound peptides were washed with 0.1% trifluoroacetic acid followed by 100 mM HEPES (pH 8). For TMTpro labeling, the lyophilized labeling reagents were dissolved in acetonitrile to a concentration of 25 μg/μl according to the manufacturer’s instructions. HEPES buffer (100 mM, pH 8) was added to a final reagent concentration of 0.5 μg/μl and the dissolved labeling reagents were passed over the bound peptides by centrifugation. The C18 resin was washed with 0.1% trifluoroacetic acid and peptides eluted with 60% acetonitrile and 0.5% acetic acid. The eluted peptides were pooled directly for each replicate. Eluates from reference samples were pooled separately and mixed into the sample replicates in equal amounts as a common reference. Peptide concentrations were measured using a NanoPhotometer N60 (Implen). A total of 75 μg peptides were centrifuged at 16 100 × *g* for 10 min. The peptide-containing supernatant was collected and evaporated by vacuum centrifugation.

### High-pH reverse-phase peptide fractionation

Peptide samples were redissolved in 30 μl solvent A (20 mM ammonium formate, pH 9.1–9.3) and centrifuged for 10 min at 16 100 × *g*. The peptides were fractionated on a Waters nanoEase M/Z Peptide CSH C18 column (300 μm × 100 mm with 130 Å, 1.7 μm C18 beads) using a Dionex Ultimate 3000 system. Peptides were loaded onto the column at 2% solvent B (80% acetonitrile in solvent A) and gradient eluted with 4–10% solvent B in 1 min, increasing to 50% solvent B in 58 min and then to 95% solvent B in 10 min, where the solvent composition was held for 6 min. In total, 50 fractions were collected and concatenated into 20 samples by combining fractions 1, 21, 41; 2, 22, 42; etc. The solvent was evaporated by vacuum centrifugation.

### Liquid chromatography–mass spectrometry

The concatenated samples were analyzed on an Easy nanoLC system coupled directly to a Thermo Fisher Orbitrap Eclipse Tribrid mass spectrometer. Peptides were redissolved in 2% acetonitrile and 0.1% trifluoroacetic acid, loaded onto a fused silica capillary column (75 μm ID, packed in-house with ReproSil-Pur C18, 1.9 μm reverse-phase material), equilibrated with solvent A (0.1% formic acid) and separated with a linear gradient of 5–35% solvent B (0.1% formic acid, 95% acetonitrile). Mass spectrometry (MS) data were acquired using synchronous precursor selection (SPS) defined by real-time database searches for accurate TMT reporter ion quantitation in MS3 spectra (SPS-MS3 method) (49). MS1 spectra were acquired in the Orbitrap using the following parameters: scan range = 400–1400; resolution = 120 000; normalized AGC target = 250%; maximum injection time = 50 ms; and RF lens = 40%. MS2 spectra were collected in the linear ion trap with the following parameter settings: data-dependent mode = cycle; time between master scan = 3; isolation window = 0.7; CID collision energy = 30%; CID activation time = 10 ms; scan rate = turbo; AGQ target = standard; and maximum injection time mode = auto. Dynamic exclusion was employed for 30 s with charge states 2–6 included for a given precursor at an intensity threshold of 1.0e4. The acquired data were subjected to real-time searches for MS3 experiments using a human UniProt filtered database and TMTpro and carbamidomethyl as static modifications and methionine oxidation as a variable modification. MS3 spectra were acquired in the Orbitrap with SPS of identified peptide fragments using the following parameters: MS1 isolation window = 0.7 *m*/*z*; MS2 isolation window = 2 *m*/*z*; HCD collision energy = 55%, Obitrap resolution = 50 000; scan range = 110–500 *m*/*z*; normalized AGC target = 500%; number of SPS precursors = 10; and data type = centroid).

The resulting data were searched in a human UniProt filtered database using MaxQuant (version 1.6.17) (50). Carbamidomethylation of cysteine residues (+57.021 Da) and TMTpro of peptide N-termini and lysine residues (304.2071 Da) were set as static modifications, while the oxidation of methionine residues (+15.995 Da) was set as a variable modification. Reporter ion intensities were adjusted to correct for neighboring isotope signals stemming from incomplete isotope incorporation and natural isotopes and then normalized between triplicate experiments and to the average of all signals for a given protein.

### 4sU RNA labeling and purification

The 4-thiouridine (4sU) RNA labeling and purification steps were carried out as described in (26), with a single modification: the input for the biotinylation reaction was 450 μg instead of 400 μg purified RNA.

### *In vitro* polyadenylation

The *in vitro* polyadenylation was carried out using *Escherichia coli* poly(A) polymerase (Invitrogen) as described in (26).

### Western blotting analysis

Harvested cells were lysed in an appropriate volume of RSB100 buffer (10 mM Tris, pH 7.4, 100 mM NaCl, 7.5 mM MgCl_2_, 0.5% Triton X-100) supplemented with protease inhibitor (cOmplete ULTRA Tablets, Roche) for 20 min at 4°C. After a centrifugation step at 12 000 × *g* for 5 min at 4°C, the protein concentration was determined by the Bradford assay (Bio-Rad). Equal amounts of protein were separated according to standard procedure using NuPAGE^®^ gels and reagents (Invitrogen) in a XCell SureLock™ Electrophoresis System. Following protein transfer from the gel onto poly(vinylidene fluoride) membranes, these were blocked in 5% milk in phosphate-buffered saline with 0.05% Tween 20 (PBS-T) for 1 h at room temperature. Membranes were subsequently incubated with primary antibody diluted in 5% milk/PBS-T either for 1 h at room temperature or at 4°C overnight (all antibodies and their dilutions are listed in Supplementary Table S4). After 3 × 10 min washes with PBS-T, the membranes were incubated with secondary antibody (diluted 1:5000 in PBS-T) for 1 h at room temperature and washed again 3 × 10 min in PBS-T. The signal was detected using SuperSignal™ West Femto Maximum Sensitivity HRP substrate (Thermo Fisher) and digitally captured using an Amersham Imager 600 (GE).

### RNA isolation and RT-qPCR analysis

RNA was isolated by TRIzol (Ambion) and treated with TURBO DNase (Ambion) according to the manufacturer’s instructions. cDNA was prepared with SuperScript III (Invitrogen) using 20 ng/μl random hexamer primer and 10 μM oligo(dT) primers. Subsequent qPCR analysis was performed with Platinum SYBR Green qPCR SuperMix-UDG (Invitrogen). All employed primers are listed in Supplementary Table S5.

### GEO datasets

RNAi 3′ end sequencing data were described in (26) and are available at Gene Expression Omnibus (GEO) under accession code GSE137612. RNA sequencing data were described in (19,29) and are available at GEO under the accession codes GSE84172 and GSE151919, respectively. AID 3′ end sequencing data have been deposited in GEO under the accession number GSE179106.

### RNA 3′ end sequencing quality control, filtering and mapping

RNA 3′ end sequencing quality control, filtering and mapping step was carried out as described in (26).

### RNA 3′ end sequencing normalization and differential expression analysis

Dataset normalization was based on the assumption that bulk mRNA levels should not be sensitive to RNA exosome depletion. Signal coverage was collected in last exons of protein-coding genes and filtered for exons with >100 reads in at least one library. After addition of a pseudocount of 1, the normalization factor of RNAi samples was calculated as described in (26), while size factors for AID samples were obtained through a related calculation using the estimateSizeFactor function within the DESeq2 package (v1.30.0) (51).

These normalization factors were employed for normalization of the respective BigWig files and for normalization of 3′ end cluster counts (see the ‘Aggregation, annotation and classification of 3′ end clusters’ section). For RNAi data, differential expression analysis was performed as described in (26), including the respective corrections for batch effects stemming from separate library preparation and sequencing dates. Such corrections were not required for AID data.

For AID data, analysis of differential expression of 3′ end cluster counts of duplicate pA^+^ and pA^+/−^ AID depletion samples relative to the respective 0 h IAA sample, the DESeq2 package (v1.30.0) (51) was employed.

### Aggregation, annotation and classification of 3′ end clusters

The 3′ end cluster coordinates from (26) were employed to aggregate also the AID 3′ end sequencing data. To assign clusters to transcript annotations, the R/Bioconductor package GenomicRanges (v1.36.1) (52) was employed to overlap cluster coordinates with HeLa-specific transcriptome annotations from (29).

### Principal component analysis

For principal component analysis (PCA), DESeq2 (v1.30.0) (51) was employed first to create a DESeq object out of the raw 3′ end cluster counts of all AID samples, second to transform the object using the DESeq2 internal function ‘vst’ (variance stabilizing transformation) and lastly to generate the PCA data frame (‘plotPCA’ function) entailing the top 2000 clusters ranked by variance across analyzed samples. This workflow was applied independently to pA^+^ and pA^+/−^ libraries and the final plots were created using the ‘ggplot’ function within the ggplot2 package (v3.3.3) (53).

### Metagene profiles

For metagene plotting, the computeMatrix tools from the deepTools software suite (v.3.3.1) (54) were combined with custom Python scripts (26). By default, metagene plots were computed in single nucleotide resolution of the normalized signal coverage and displayed as log_2_ ratios between the individual samples and the respective controls (for AID, the 0 h IAA HeLa:TIR1 sample; for KD, the siEGFP sample). Before log transformation, a pseudocount of 1 was added to all values.

Employed annotations were generally taken from (29), while branchpoint annotations stem from (55) and snoRNA annotations from Gencode, release 36 (GRCh38).

### G-quadruplex predictions

For individual G-quadruplex prediction, QGRS Mapper (56) was employed.

### Genome track views

Sequencing tracks were displayed in R using a plotting pipeline developed by Dr Søren Lykke-Andersen (manuscript in preparation).

### Exosome sensitivity values

For estimation of the sensitivity to the given depletion condition, the following formula was employed: sensitivity = (treated − ctrl)/max(ctrl, treated, 1). For AID- and RNAi-mediated depletions, the HeLa:TIR1, 0 h IAA and the siEGFP sample acted as respective control samples. Equal 3′ end signal in treated and ctrl samples returns a sensitivity value of 0, while signal exclusive to the depletion or 0 h control sample corresponds to a sensitivity value of 1 and −1, respectively.

### Differential protein expression

After removal of all proteins labeled as ‘potential contaminant’ or ‘reverse’, analysis of differential expressed proteins was performed in R using the DEP package (v1.12.0) (57). Proteins identified in all replicates of at least one condition were filtered (‘filter_missval’ function, with settings: thr = 0) and the values were normalized by variance stabilizing transformation (‘normalize_vsn’ function). Missing values were imputed by random draws from a Gaussian distribution centered to a minimal value (‘impute’ function with settings: fun = ‘MinProb’, *q* = 0.01). Differential enrichment testing (‘test_diff’ function) was based on manually set contrasts (condition versus unedited reference) and followed the following design: formula(∼0 + condition + replicate), where condition refers to the tagged protein plus the IAA time point information. Proteins with adjusted *P*-values (*P*_adj_) < 0.05 were considered significant.

Significant proteins with log_2_ fold change (log_2_FC) < −1 or log_2_FC > 1 in at least one sample were isolated from all samples and subjected to *k*-means clustering within the ‘plot_heatmap’ function with settings (type = ‘contrast’, *k*-means = TRUE, *k* = 6, plot = F).

### GO analysis

Gene Ontology (GO) annotations were based on protein IDs and retrieved using the ‘biomaRt’ package (v2.46.2) (58,59). Individual enrichment analysis of biological process annotation (ontology = ‘BP’) was performed using the package ‘topGO’ (v2.42.0) (60), employing ‘weight01’ algorithm and 'Fisher' statistic settings on proteins within *k*-means protein clusters (see the ‘Differential protein expression’ section) versus all proteins identified in the MS analysis. GO terms with <5 annotated genes were removed from the analysis (nodeSize = 5). After BH correction on the obtained *P*-value, GO terms with *P*_adj_ ≤ 0.05 were considered significant and the top 5 most significant terms within each cluster were displayed using ggplot2.

## RESULTS

### Rapid depletion of ZCCHC8, ZFC3H1 and EXOSC3

**Figure 1.**
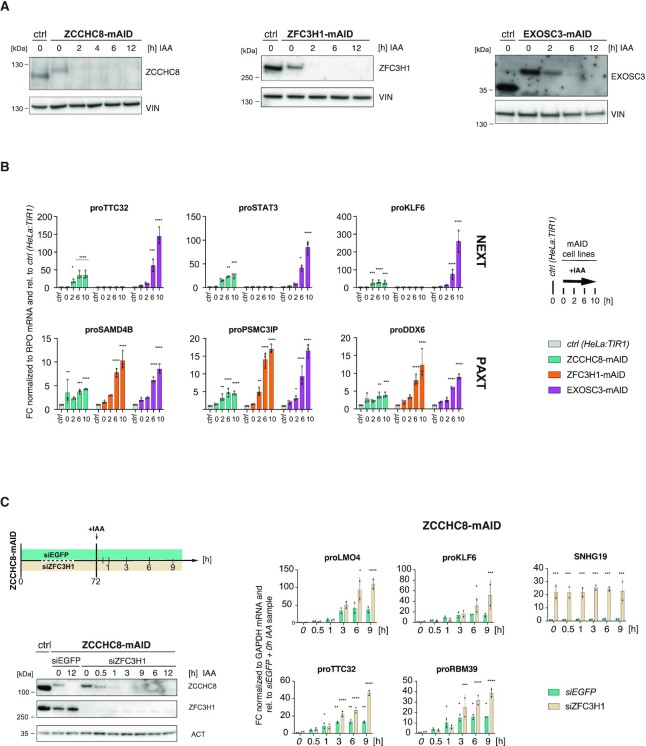
A rapid depletion strategy provokes adaptor-specific substrate responses. (**A**) Western blotting analysis of unedited (ctrl) together with edited HeLa:TIR1 ZCCHC8-mAID (left panel), ZFC3H1-mAID (middle panel) and EXOSC3-mAID (right panel) cells. Degron-tagged cell lines were exposed to 750 μM IAA for the indicated time periods (h). Blots were probed with endogenous antibodies against ZCCHC8, ZFC3H1, EXOSC3 and vinculin (VIN) as a loading control. (**B**) RT-qPCR of total RNA analyzing three NEXT (top panel) and three PAXT (bottom panel) PROMPTs from depletion time course experiments analogous to (A). Values were normalized to Rplp0 (RPO) mRNA levels and plotted relative to results from unedited, non-IAA-treated HeLa:TIR1 (ctrl) cells (see italics). Columns represent average values of biological triplicate samples with error bars denoting standard deviations. Individual data are indicated as points. Significance of the difference between a given time point and the untreated control sample was determined by a two-way ANOVA test (*P_adj_ ≤ 0.05, **P_adj_ ≤ 0.01, ***P_adj_ ≤ 0.001, ^****^P_adj_ ≤ 0.0001, nonsignificant FC with *P*_adj_ > 0.05 is not marked). (**C**) Western blotting (left panel) and RT-qPCR (right panel) analyses as in (A) and (B) but of ZCCHC8-mAID cells pretreated with siRNA against ZFC3H1 (siZFC3H1) or an siRNA control (siEGFP) and subjected to the indicated IAA time course (upper left schematics). Left panel: Actin (ACT) was used as a loading control. Right panel: Results are shown relative to the siEGFP + 0 h IAA sample (see italics) and normalized to GAPDH mRNA levels. Amplified regions include four NEXT PROMPTs (left) and, to confirm ZFC3H1 depletion, one PAXT target (SNHG19, right). Display and significance values as in (B), except that data were from biological duplicate samples.

To create a cell system for the rapid depletion of nuclear exosome (co)factors, we used CRISPR/Cas9 genome editing to introduce a truncated version of the AID tag (mAID) (43) at the C-termini of the endogenous ZCCHC8, ZFC3H1 and EXOSC3 proteins in TIR1-expressing HeLa cells (Supplementary Figure S1A). Stable homozygous integration was confirmed at both the DNA (Supplementary Figure S1B) and protein (Figure 1A) levels. Consistent with previous instances of AID tagging, fusion proteins were slightly underexpressed compared to their endogenous counterparts even in the absence of auxin (Figure 1A) (10,61,62). Nevertheless, IAA time course experiments demonstrated that these new baselines of expression could be rapidly depleted, reducing proteins levels to below western blotting detection limits within 2 h for ZCCHC8-mAID and ZFC3H1-mAID, and 6 h for EXOSC3-mAID (Figure 1A). Together with undisturbed proliferation within these time frames (data not shown), successful protein depletions encouraged further validation of the mAID cell lines at the level of nuclear exosome substrates.

To examine the functional consequences of factor depletion, total RNA was isolated from depletion time points and analyzed by RT-qPCR for individual PROMPT substrates of NEXT (proTTC32, proSTAT3, proKLF6; Figure 1B, upper panel) and PAXT (proSAMD4B, proPSMC3IP, proDDX6; Figure 1B, lower panel) decay pathways. Compared to the unedited cell line (‘ctrl’), the slightly reduced basal levels of the tagged proteins in the absence of IAA were associated with a modest accumulation of the tested RNAs. In contrast, inducing protein depletions (2–10 h + IAA samples) led to drastic substrate RNA accumulation, corroborating predicted NEXT and PAXT adapter specificities (Figure 1B). Of note, PAXT-sensitive transcripts increased slightly upon depletion of ZCCHC8, which likely reflects a production of nonadenylated transcript 3′ end isoforms within PAXT-sensitive TUs (26). Finally, substrate levels were not altered in ctrl cells exposed to IAA (Supplementary Figure S1C), confirming that transcript accumulation specifically occurred as a consequence of targeted protein removal but not of auxin treatment.

Concurrent with continuous protein depletion, NEXT- and PAXT-sensitive transcripts were expected to progressively accumulate. Such steady increases in substrate levels could indeed be observed upon depletion of ZFC3H1 and EXOSC3, whereas ZCCHC8-dependent substrates plateaued at new steady-state levels after 2–6 h IAA exposure (Figure 1B, compare green, orange and purple columns). As NEXT substrates can undergo 3′ end adenylation and consequently become available for PAXT-mediated decay (26), we considered whether this limited accumulation could be a consequence of such substrate handover from NEXT to PAXT. Therefore, we measured NEXT substrate levels upon IAA-mediated depletion of ZCCHC8-mAID in cells, which were subjected to siRNA-mediated depletion of ZFC3H1 (Figure 1C, left panel). In this double depletion experiment, NEXT substrates no longer plateaued, but progressively accumulated throughout the time course (Figure 1C, right panel), strongly supporting the concept of a fail-safe decay of NEXT substrates via PAXT-mediated RNA targeting.

We conclude from the specific accumulation of known RNA targets that IAA-induced rapid depletion of ZCCHC8-, ZFC3H1- and EXOSC3-mAID allows to probe the endogenous functions of NEXT, PAXT and the exosome in general. In addition, the rapid degradation adds a previously inaccessible kinetic resolution, enabling a more detailed insight into NEXT and PAXT biology.

### Proteome effects upon factor depletions

**Figure 2.**
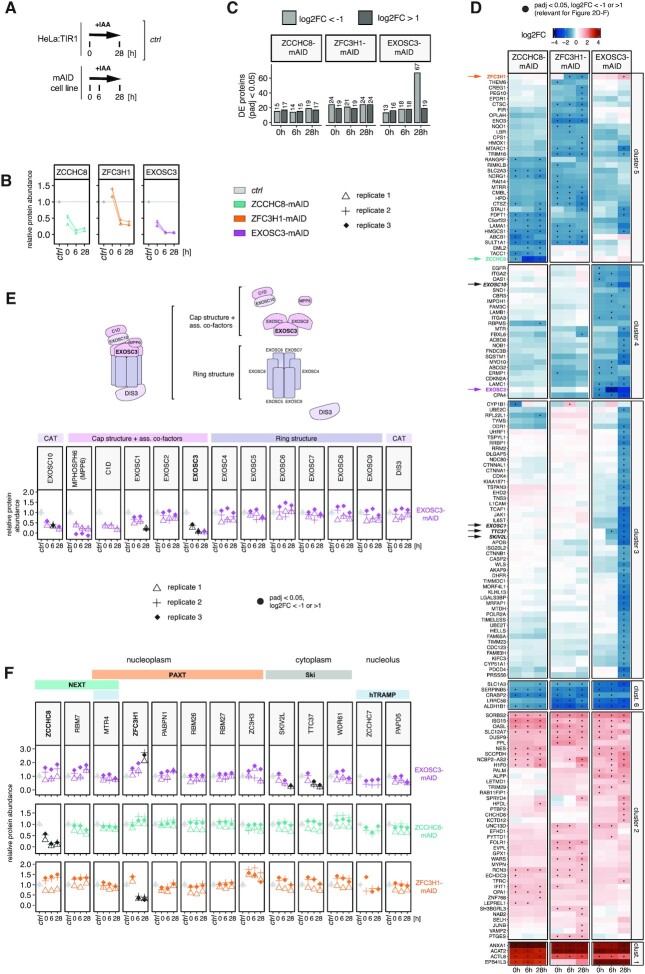
Proteome effects upon factor depletions. (**A**) Schematic outline of conditions employed for proteomic analyses. ZCCHC8-, ZFC3H1- and EXOSC3-mAID cells (‘mAID cell lines’) were exposed to IAA for 0, 6 and 28 h. All whole cell MS data were analyzed relative to a control ('ctrl’), constituting a merge of the 0 and 28 h IAA treatment samples from unedited HeLa:TIR1 cells. (**B**) Relative abundances of ZCCHC8, ZFC3H1 and EXOSC3 in their respective mAID-tagged cell lines exposed to IAA for the indicated time points (0, 6 and 28 h). Biological triplicate experiments are displayed individually. (**C**) Bar plots showing numbers of significantly (*P*_adj_ < 0.05) altered proteins, according to differential protein expression analysis (see the ‘Materials and Methods’ section), from ZCCHC8-, ZFC3H1- and EXOSC3-mAID depleted samples harvested at the indicated time points. Absolute numbers are indicated above each bar. (**D**) Clustered heatmap representations (*k*-means, *k* = 6) of log_2_FC upon IAA exposure for the indicated time points in ZCCHC8-, ZFC3H1- and EXOSC3-mAID cell lines. All proteins significantly (*P*_adj_ < 0.05) up- or downregulated with log_2_FC < −1 or log_2_FC > 1 in at least one sample are displayed. Black dots indicate instances that match the said significance criteria. (**E**) Relative protein abundances of RNA exosome subunits in EXOSC3-mAID cells. Top panel: Schematic representation of the 13-subunit nuclear RNA exosome subdivided into catalytic subunits (CAT), the cap structure including associated cofactors and the ring structure. Bottom panel: Proteomic analyses of the above proteins during IAA-mediated depletion of EXOSC3 as indicated at the *x*-axes. Protein changes considered to be significant (*P*_adj_ < 0.05) and with a log_2_FC relative to the control of <−1 or >1 are displayed in black. (**F**) Relative abundances of RNA exosome-related proteins in all of the mAID-tagged cell lines (EXOSC3: top; ZCCHC8: middle; ZFC3H1: bottom). Shown proteins compose RNA exosome-associated adaptor complexes in the nucleoplasm (NEXT and PAXT), the cytoplasm (SKI complex) and the nucleolus (hTRAMP). Statistical analysis as in (E).

To assess global proteomic changes, including secondary effects, emerging as a result of mAID-mediated depletion of ZCCHC8, ZFC3H1 and EXOSC3, we interrogated samples after 0, 6 or 28 h of depletion by MS (Figure 2A). A total of 6500 proteins were quantified in all conditions in triplicate experiments. As expected, all mAID-tagged proteins were significantly depleted upon IAA treatment (Figure 2B). Consistent with our western blotting analysis, we also observed lower basal levels of ZCCHC8-mAID and EXOSC3-mAID in untreated cells relative to the unedited control samples (Figure 2B, compare with Figure 1A). The MS data did, however, not corroborate lowered basal protein levels for ZFC3H1-mAID, despite its clear decline after 6 and 28 h of IAA treatment. We suspect that the MS data may reflect protein fragments that are not visible on the immunoblot.

We next applied differential expression analysis, comparing proteomes from the tagged cell lines to the untagged control, in the absence and presence of auxin. Across all samples, we observed in total 158 significantly affected proteins, which we grouped by *k*-means clustering into six clusters with similar expression patterns in response to exosome, NEXT and PAXT depletion (Figure 2C and Supplementary Table S6). This analysis highlighted that, compared to the targeted proteins, most changes were visible before IAA treatment and often shared between samples (Figure 2D, black dots represent *P*_adj_ < 0.05 and log_2_FC > 1 or log_2_FC < −1). A notable exception was cluster 3, comprised mostly of proteins exclusively downregulated following long-term EXOSC3 depletion. This cluster was associated with GO terms related to cell division and cell cycle progression, possibly reflecting a cell growth deficiency in this condition, which manifested upon prolonged exposure (>24 h) to auxin, presumably due to the essential function of the RNA exosome (Supplementary Figure S2A and data not shown) (10,63–66). In contrast, cluster 2 was dominated by upregulated proteins and enriched for factors linked to viral defense mechanisms and innate immune responses (Figure 2D and Supplementary Figure S2A), indicating that accumulation of NEXT/PAXT/exosome RNA substrates triggers cellular defense pathways. Finally, since clusters 1 and 2 comprised upregulated proteins, we initially suspected that these might be caused by concomitant increases in the associated mRNAs (Figure 2D). However, RNA sequencing data from RNAi depletion samples (19) did not confirm such upregulation (Supplementary Figure S2B, black dots represent *P*_adj_ < 0.1 and log_2_FC > 1 or log_2_FC < −1). Conversely, and with a sole exception, previously identified exosome-sensitive mRNAs (67) did not give rise to upregulated proteins (data not shown). This suggests that these accumulated transcripts are either poorly exported to the cytoplasm or, perhaps less likely, not efficiently translated.

With the exception of the prolonged depletion of EXOSC3, our strategy for mAID depletion of exosome, NEXT and PAXT appeared to minimize secondary alterations of the proteome. That said, several established exosome-related proteins were conspicuously up- or downregulated. More specifically, induction of EXOSC3 degradation led to co-depletion of EXOSC10, MPHOSPH6 (MPP6), C1D and EXOSC1, which are all in close physical proximity to EXOSC3 in the upper exosome cap structure (Figure 2E). Co-depletion was not observed for EXOSC2 and for all subunits within the lower exosome ring structure (Figure 2E and Supplementary Figure S2C), indicating, somewhat unexpectedly, that the RNA exosome is organized in submodules with distinct post-translational regulation. Additionally, the TTC37 and SKIV2L components of the cytoplasmic superkiller (SKI) adaptor complex, consisting of the scaffold protein TTC37, two copies of WDR61 and the helicase SKIV2L (68,69), also displayed significant co-depletion along with EXOSC3. In contrast, none of the nuclear adaptor components from NEXT, PAXT and hTRAMP were significantly negatively affected (Figure 2F, upper panel), which implies a stronger reliance of SKI complex stability on the exosome core/cap. In fact, ZFC3H1 levels were increased upon EXOSC3 depletion (Figure 2F, upper panel), which could only be replicated to lesser extent by western blotting analysis (Supplementary Figure S2C). While this could partially be explained by the slight, yet nonsignificant, upregulation of ZFC3H1 mRNA (Supplementary Figure S2B and D), altered protein stability in response to EXOSC3 depletion cannot be ruled out. Any other changes in protein levels were not mirrored by respective changes at the mRNA levels (Supplementary Figure S2B and D). Finally, and consistent with earlier RNAi studies (70–72), depletion of ZCCHC8 was mirrored by decreased RBM7 protein (Supplementary Figure S2E and Figure 2F, middle panel), not mRNA levels (Supplementary Figure S2F), whereas ZFC3H1 depletion caused increased protein levels of the PAXT component ZC3H3 (Figure 2F, bottom panel). These opposing effects may reflect the tight ZCCHC8–RBM7 interaction, whereas the weaker physical connection between ZFC3H1 and ZC3H3 may allow for compensatory protein upregulation (18,22). We also note that MTR4 was only mildly decreased in either of the ZCCHC8 and ZFC3H1 depletions, likely due to its engagement in multiple additional complexes that may preserve its stability (73).

We conclude that our experimental system allowed us to deplete mAID-tagged factors rapidly and completely, without significant proteomic changes outside of direct interactors. That said, prolonged absence of key turnover factors, such as EXOSC3, exposed altered protein levels, likely caused by perturbed cell physiology, and advocating for the use of short-time factor depletion.

### Rapid factor depletion reveals direct nucleoplasmic exosome targets

**Figure 3.**
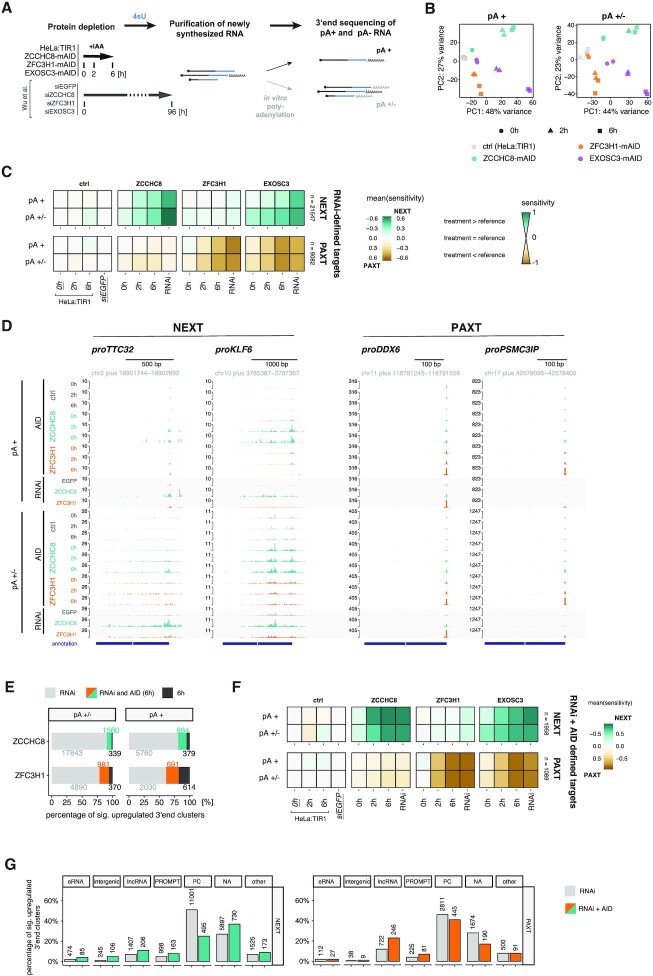
AID-mediated depletion enables identification of direct targets of NEXT and PAXT. (**A**) Experimental workflow for 4sU RNA 3′ end sequencing sample preparation. Factor depletion was followed by 4sU addition and purification of 4sU-labeled RNA. An *in vitro* polyadenylation step, or absence thereof, enabled detection of both pA^+^ and pA^−^ RNA species. Transcriptome changes upon AID-mediated protein depletions were compared to those from ‘RNAi’ datasets (26). (**B**) PCA of the generated pA^+^ and pA^+/−^ 3′ end sequencing libraries from unedited HeLa:TIR1 (ctrl, gray), ZCCHC8-mAID (green), ZFC3H1-mAID (orange) and EXOSC3-mAID (purple) cells upon exposure to IAA for 0 h (circle), 2 h (triangle) or 6 h (square). PCA was computed with the top 2000 3′ end clusters ranked by variance across all samples. (**C**) Sensitivity of RNA 3′ end clusters within previously defined RNAi-based NEXT (upper panel) and PAXT (lower panel) targeting categories (26). Sensitivity values, ranging from −1 to 1, reflect the relationship between the 3′ end signal in the displayed samples relative to the respective reference sample (for AID: HeLa:TIR1, 0 h IAA; for RNAi: siEGFP). Positive values correspond to higher signal in the depletion than the reference sample, with signal exclusive to the depletion sample resulting in a sensitivity value of 1 (vice versa for negative values). Equal expression in depletion and reference corresponds to a sensitivity value of 0 (for details, see the ‘Materials and Methods’ section). (**D**) Genome browser views of selected NEXT (proTTC32, proKLF6) and PAXT (proDDX6, proPSMC3IP) target regions, visualizing 3′ end data from AID (0, 2 and 6 h) and RNAi depletion conditions. Upper and lower panels show pA^+^ and pA^+/−^ libraries, respectively. Data from HeLa:TIR1/siEGFP ctrl, ZCCHC8 depletion and ZFC3H1 depletion samples are shown in gray, green and orange, respectively. Tracks are group scaled within pA^+^ and pA^+/−^ libraries. (**E**) Intersection of upregulated (log_2_FC > 1, *P*_adj_ < 0.1) pA^+^ and pA^+/−^ 3′ end clusters upon 6 h IAA- or RNAi-mediated depletion of ZCCHC8 and ZFC3H1 relative to the respective reference sample (AID: 0 h IAA; RNAi: siEGFP; see Supplementary Figure S3A for a schematic overview of differential expression analysis). Absolute numbers are displayed next to the respective fraction. (**F**) Sensitivity maps as in (C) but showing mutually exclusive RNAi + AID NEXT and PAXT targeting classes based on the intersected ‘RNAi and AID (6 h)’ 3′ end clusters from (E). (**G**) Bar plots showing the percentage of 3′ end clusters within RNAi- and RNAi + AID-based NEXT and PAXT targeting classes that overlap with the indicated RNA biotypes. Respective absolute numbers of 3′ end clusters are included on top of the individual bars. PC: protein coding; NA: not annotated.

Encouraged by the minimal proteomic effects upon short-term factor depletions, we proceeded to investigate transcriptome-wide, depletion-induced changes. Our previous RNAi-based study demonstrated the importance of RNA 3′ end status for NEXT/PAXT-mediated decay mechanisms (26), prompting a 3′ end sequencing approach to elucidate the positioning and polyadenylation status of NEXT- and PAXT-sensitive transcript termini (Figure 3A). In brief, time course depletion of ZCCHC8-, ZFC3H1- and EXOSC3-mAID (0, 2 and 6 h) and unedited HeLa:TIR1 cell lines was followed by metabolic labeling with 4sU for 10 min. Newly synthesized 4sU RNA was purified and used either directly or after *in vitro* polyadenylation with *E. coli* poly(A) polymerase, for adenylation-based 3′ end RNA sequencing. This captured either solely *in vivo* adenylated transcripts (pA^+^) or the combination of adenylated and nonadenylated RNAs (pA^+/−^), in directly comparable datasets, which were similar to analogously generated data from RNAi samples (Figure 3A) (26).

For global analysis of the sequencing data, we aggregated proximal 3′ end positions into ‘3′ end clusters’ (26). PCA of normalized read counts within these clusters revealed adaptor- and time point-specific substrate targeting profiles (Figure 3B). Reassuringly, ZCCHC8 and ZFC3H1 depletion datasets were separated already from the earliest time point (2 h) of protein depletion, while EXOSC3 datasets were positioned between these two extremes, in accordance with the exosome functioning in both targeting pathways. This clear distinction motivated us to investigate how NEXT- and PAXT-dependent transcriptome changes would compare between short-term (AID) and long-term (RNAi) protein depletion conditions. Hence, we assessed the sensitivity of 3′ end clusters to factor depletion within mutually exclusive PAXT and NEXT targeting classes that were previously defined by RNAi-mediated factor depletion (Figure 3C, ‘RNAi-defined targets’) (26). Our new AID data confined to these mutually exclusive NEXT and PAXT sensitivities and showed increased sensitivity, and thus target accumulation, within the NEXT targeting class upon ZCCHC8 depletion and within the PAXT targeting class upon ZFC3H1 depletion. As expected, both substrate categories responded to EXOSC3 depletion (Figure 3C). Notably, sensitivity values ensuing AID-mediated depletion were overall smaller than those resulting from RNAi-mediated depletion, which was further reflected by fewer significantly upregulated (*P*_adj_ < 0.1, log_2_FC > 1) 3′ end clusters (Supplementary Figure S3A). However, RT-qPCR assessment of individual substrates from RNAi- or AID-mediated depletion samples still revealed elevated (ZCCHC8), or comparable (ZFC3H1), substrate accumulation in total RNA fractions (Supplementary Figure S3B) and was consistent with similar protein depletion efficiencies between the two respective approaches (Supplementary Figure S3C). Moreover, corresponding 4sU sequencing tracks, displaying example substrates, did not support a generally stronger RNAi phenotype (Figure 3D, proKLF6 and proPSMC3IP). Taken together, this motivated a more detailed comparison of exactly which RNAs accumulate after short- versus long-term depletion.

To address this question, we first ensured that the AID- and RNAi-generated datasets were sequenced at similar depths (Supplementary Figure S3D). Interestingly, however, comparing targets upregulated in ZCCHC8 RNAi versus 6 h AID depletion samples revealed a clear difference in basal expression levels (Supplementary Figure S3E). That is, RNAi depletion of ZCCHC8 led to the accumulation of a large set of lowly expressed pA^+/−^ 3′ ends, which did not appear in the corresponding AID depletion samples. We reasoned that this reflected indirect effects and thus used the AID depletion data to more precisely define primary NEXT and PAXT targets. To this end, we intersected pA^+^ and pA^+/−^ 3′ end clusters upregulated in both of the 6 h AID- and RNAi-mediated ZCCHC8 or ZFC3H1 depletion samples [Figure 3E, ‘RNAi and AID (6 h)’]. The selected 3′ end clusters were then classified based on their exclusive targeting by either ZCCHC8 or ZFC3H1, resulting in new classes of NEXT- and PAXT-sensitive 3′ end clusters (‘RNAi + AID defined targets’), which displayed the expected upregulation in both the RNAi and the 6 h AID samples (Figure 3F). Upon feature comparison of these stringent ‘RNAi + AID defined targets’ to their ‘RNAi’ counterparts, well-established nuclear exosome substrates, such as eRNAs, lncRNAs and PROMPTs, became better represented (Figure 3G). In contrast, 3′ ends from within protein-coding genes were more frequent in the RNAi-based ZCCHC8 samples, accounting for up to 50% of 3′ end clusters within this substrate category (Figure 3G). No strong biotype distinction was observed between the RNAi and ‘RNAi + AID (6 h)’ ZFC3H1 samples, corroborating the less distinct phenotypes between long- and short-term depletion in this case.

We conclude that rapid depletion of ZCCHC8 provokes a more stringent transcriptome alteration than its long-term depletion, which instead accumulated excessive 3′ ends within protein-coding genes, possibly as an indirect consequence of long-term absence of NEXT.

### Intron decay defects are exacerbated upon long-term absence of NEXT

**Figure 4.**
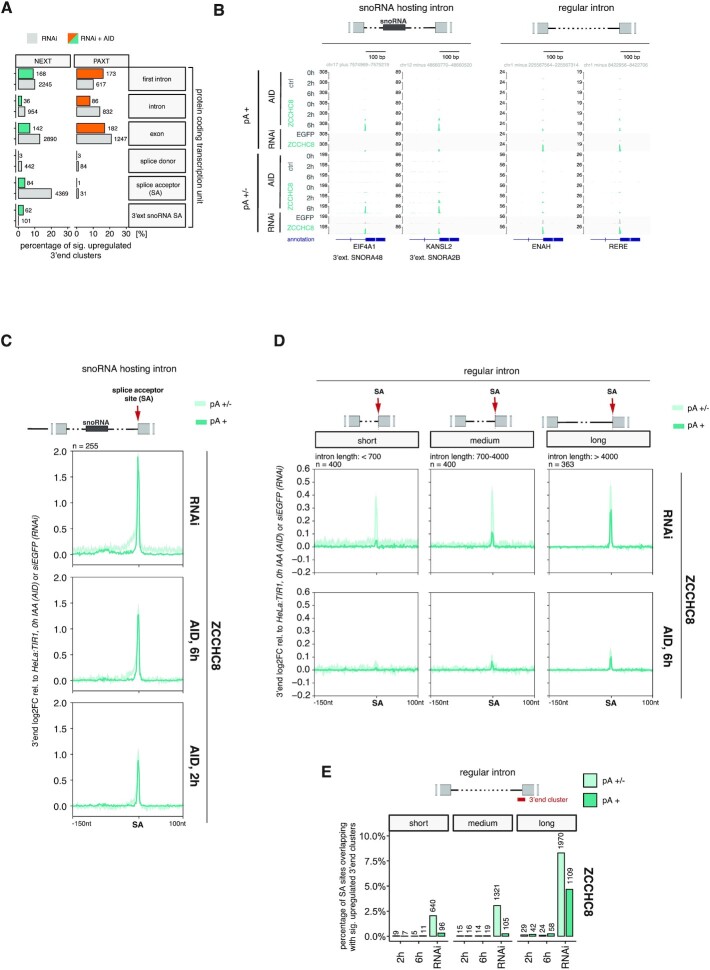
Rapid ZCCHC8 depletion establishes NEXT targeting of snoRNA-hosting introns. (**A**) Bar plots showing the percentage of 3′ end clusters within ‘RNAi’- and ‘RNAi + AID’-based NEXT and PAXT targeting classes that overlap with the indicated features within protein coding TUs. Respective absolute numbers of 3′ end clusters are included next to the individual bars. (**B**) Genome browser views of selected SAs of snoRNA-hosting (left) or regular (right) introns, showing data from AID- (0, 2 and 6 h) and RNAi-mediated ZCCHC8 depletion samples. pA^+^ and pA^+/−^ libraries are displayed as in Figure 3D. HeLa:TIR1 and siEGFP ctrl samples were included as controls. (**C**) Metagene profiles of the indicated regions up- and downstream of SA sites of snoRNA-hosting introns (see schematics on top). Values shown are log_2_FC in single nucleotide resolution between pA^+^ and pA^+/−^ 3′ end-seq RNAi or 6 h AID libraries following ZCCHC8 depletion relative to their respective controls (RNAi: siEGFP; AID: HeLa:TIR1, 0 h IAA). The displayed region covers 150 nt upstream and 100 nt downstream the SA anchor point. (**D**) Metagene profiles as in (C), but of regular introns in otherwise snoRNA-hosting transcripts and stratified into three different classes, according to intron length (left: <700 bp; middle: 700–4000 bp; right: >4000 bp). (**E**) Bar plots showing the percentage of SA sites of short, medium and long introns [see panel (D)], overlapping with significantly upregulated 3′ end clusters in ZCCHC8 depletion conditions (2 h, 6 h, RNAi) relative to the respective controls (AID: 0 h IAA; RNAi: siEGFP). Absolute numbers of SA sites are included on top of the individual bars.

To pinpoint exact features of the direct versus indirect NEXT-targeted 3′ ends within protein-coding genes, we stratified the positions of upregulated 3′ end clusters to gene-specific regions/anchor points relevant for nuclear exosome targeting (Figure 4A) (24–26,29,34). As expected, only moderate differences were observed between the ‘RNAi’ and ‘RNAi + AID (6 h)’ ZFC3H1 samples. In contrast, ZCCHC8 depletion samples revealed a major difference for 3′ ends mapping to splice acceptor (SA) sites, which contributed ∼20% of all significantly upregulated clusters in the ‘RNAi’ targeting class but only ∼5% in the stringent ‘RNAi + AID’ class. A noticeable exception was SA sites of snoRNA-hosting introns, which appeared more frequently in the ‘RNAi + AID’ data (Figure 4A). In fact, these 3′ ends became observable NEXT targets already upon 2 h of AID-mediated ZCCHC8 depletion (Figure 4B and C), confirming their status as RBM7-bound primary RNA exosome substrates (24,34).

The contribution of NEXT-mediated targeting of regular introns was previously suggested based on RBM7 CLIP binding profiles and physical connections between NEXT and splicing factors (24,35). Since our data implied that this could only be functionally verified upon long-term NEXT depletion (Figure 4A and B), we examined general SA site targeting and intron degradation in further detail. Upon their splicing and debranching, intronic sequences expose unprotected 5′ and 3′ ends, which become available for 5′–3′ and 3′–5′ exonucleases, respectively. As exonucleolytic decay kinetics are at least somewhat proportional to substrate length, we first stratified introns by their length and also compared regular introns to their snoRNA-hosting counterparts. To not bias our analysis, we ensured similar expression levels by focusing on regular introns within snoRNA host transcripts. Metagene analysis of the respective SA sites (Figure 4D), as well as their overlap with significantly upregulated 3′ end clusters (Figure 4E), displayed a clear positive correlation between 3′ end accumulation and intron length, especially upon RNAi-mediated depletion. The increased sensitivity of SA sites to ZCCHC8 depletion with increasing intron length was particularly evident for the ‘RNAi’ pA^+^ 3′ end data (Figure 4D and Supplementary Figure S4A). Given that adenylation of pA^−^ 3′ ends occurs in the absence of NEXT-mediated decay (26), the pA^+^ state distinguished these 3′ end clusters from true splicing intermediates captured by the 4sU labeling approach. Metagene analysis of total RNA samples further corroborated that the accumulation of these pA^+^ RNA species was due to inefficient turnover (Supplementary Figure S4B). Such length-dependent, and largely RNAi-specific, NEXT sensitivity was observed for regular introns within and outside of snoRNA-hosting transcripts (Figure 4B and Supplementary Figure S4A and S4C), but not for snoRNA-hosting introns (compare Figure 4E and Supplementary Figure S4D). Thus, in addition to snoRNP formation, intron length appears to dictate 3′ end accumulation. As both parameters are related to the efficiency by which 5′–3′ exonucleolysis will remove the full intron, we speculate that the pronounced RNAi-specific phenotype is elicited by deficient 5′–3′ exonucleolysis upon long-term depletion of ZCCHC8/NEXT. We note that such deficiency is presumably not due to generally lowered protein levels of 5′–3′ exonucleases or proteins associated with splicing although an impact from the altered abundance of individual factors cannot be ruled out (Supplementary Figure S4E and Figure 2C).

Taken the data together, we surmise that NEXT-mediated intron degradation is primarily detected at the 3′ ends of snoRNA-hosting introns. The correlation of NEXT sensitivity with intron length suggests that regular intron turnover is largely conducted by 5′–3′ exonucleolysis. Consequently, the indirect NEXT depletion phenotype, observed upon long-term ZCCHC8 removal, indicates a concomitant decline in 5′–3′ decay activity and implies that NEXT-mediated RNA decay is likely required to complement and prevent exhaustion of available 5′–3′ exonucleolytic activity.

### NEXT-sensitive 3′ ends can be targeted for 3′–5′ exonucleolysis in the absence of EXOSC3

**Figure 5.**
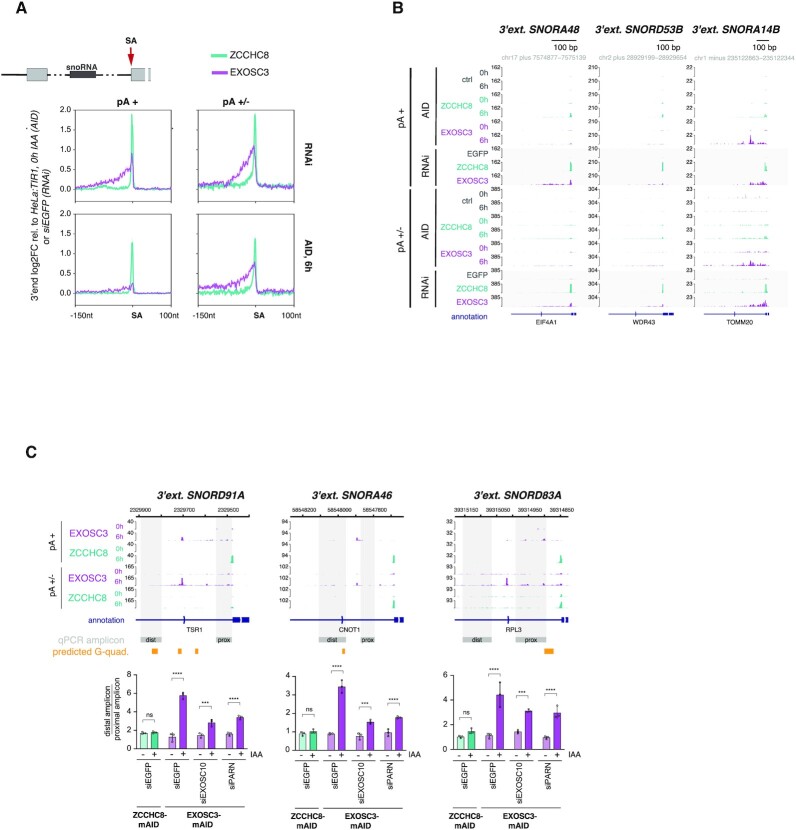
3′ end trimming in EXOSC3 depletion conditions. (**A**) Metagene profiles of regions around SA sites of intron-hosted snoRNAs, showing log_2_FC in single nucleotide resolution between pA^+^ and pA^+/−^ 3′ end sequencing data of newly synthesized RNA upon RNAi- or 6 h AID-mediated ZCCHC8 and EXOSC3 depletion relative to siEGFP or unedited HeLa:TIR1, 0 h IAA controls. Regions are displayed as in Figure 4C. (**B**) Genome browser views as in Figure 3D, but showing regions around selected SAs of snoRNA-hosting introns. Shown data are from AID- (0 and 6 h) or RNAi-mediated ZCCHC8 and EXOSC3 depletion conditions. (**C**) Trimming assay based on RT-qPCR analysis of selected amplicons (gray areas and ‘qPCR amplicon’ annotations in genome browser view) within 3′ extended snoRNAs. RT-qPCR analyses were conducted on total RNA isolated from EXOSC3-mAID (purple) and ZCCHC8-mAID (green) cells treated with siRNAs against EGFP, EXOSC10 or PARN and followed by 0 h (−) or 6 h (+) exposure to IAA. Data were normalized to Rplp0 mRNA (RPO) and displayed as the trimming factor, obtained from the ratio of distal/proximal amplicon values. Differences within RNAi treatment groups, comparing +/− IAA samples, were determined by a two-way ANOVA test [*P_adj_ ≤ 0.05, **P_adj_ ≤ 0.01, ***P_adj_ ≤ 0.001, ^****^P_adj_ ≤ 0.0001, nonsignificant (ns) = *P*_adj_ > 0.05].

Although snoRNA-hosting intron 3′ ends are targeted by NEXT and the RNA exosome (24,26,74,75), we nevertheless noticed that EXOSC3 depletions still displayed a heterogeneous distribution of 3′ ends upstream of these SA sites, which was apparent at both individual and metagene levels and in stark contrast to distinct SA 3′ end signals produced by ZCCHC8 depletion (Figure 5A and B). Closer examination of these scattered 3′ ends upon EXOSC3 depletion revealed a predominant accumulation between the SA site and the upstream snoRNA, with a positional bias toward a region ∼50 nt upstream the SA site (Supplementary Figure S5A, left panels), but with no discernable link to the positions of intronic branch points (Supplementary Figure S5A, right panels). Given their heterogeneity, we reasoned that these 3′ ends were likely a result of nonprocessive exonucleolytic trimming. Without detailed investigation, we further assumed that RNA structures such as the occasionally observed presence of a G-quadruplex (Figure 5C), nucleotide biases and/or protein binding sites all contribute to obstructing exonucleolysis.

Wondering which exonuclease(s) may be responsible for the observed EXOSC3-independent RNA trimming, we next queried the possible actions of EXOSC10, which was previously suggested to act independent of the RNA exosome core (66,76,77), and PARN, a ribonuclease targeting mostly pA stretches and previously shown to partake in the trimming of intron-encoded snoRNAs (78,79). Using RNAi, we depleted both of these nucleases in conjunction with IAA-mediated depletion of either EXOSC3 or ZCCHC8 (Supplementary Figure S5B). The extent of 3′ end trimming was assessed by RT-qPCR analysis of total RNA, using amplicons positioned either proximal or distal to the SA site (Figure 5C, see gray ‘dist’ and ‘prox’ boxes for amplicon positioning). As expected, ZCCHC8 depletion led to an equal signal increase for both the proximal and distal amplicons, reflecting the total absence of trimming (Figure 5C, green bars). In contrast, EXOSC3 depletion yielded a stronger signal by the SA-distal amplicon (Figure 5C, siEGFP, purple bars), validating the 3′ end trimming phenotype from our sequencing data. This difference between SA-distal and -proximal amplicon signals was reduced upon additional depletions of both EXOSC10 and PARN (Figure 5C, purple bars). The suppression was due to more efficient amplification of the proximal region (Supplementary Figure S5C, left panel), which altogether indicated an active role of both nucleases in nonprocessive RNA trimming in the absence of EXOSC3. Notably, the distal targeting region showed an additive accumulation in co-depletion conditions, hinting toward an alternative targeting mode of these 3′ ends in the absence of their primary decay pathway. This scenario was further supported by a similar additive signal observed upon co-depletion of EXOSC10 or PARN with ZCCHC8 (Supplementary Figure S5C, right panel).

Is EXOSC3-independent trimming restricted to snoRNA-hosting introns or is it more general? To address this question, we first interrogated SA upstream regions of long introns, which previously demonstrated to display NEXT sensitivity in RNAi depletion conditions (Figure 4D). Comparative metagene analysis of ZCCHC8 and EXOSC3 depletion samples revealed an overall lower and more scattered patterning of 3′ ends in the EXOSC3 dataset (Supplementary Figure S5D). Second, we focused on ZCCHC8-sensitive 3′ ends arising following miRNA processing. Here, the upstream product of endonucleolytic cleavage by the microprocessor complex was previously demonstrated to be targeted by NEXT (24,80). Global analysis of the AID and RNAi 3′ seq datasets confirmed this notion and further revealed EXOSC3-independent 3′ end trimming of these targets (Supplementary Figure S5E).

Taken together, we conclude that NEXT-sensitive 3′ ends can be trimmed in both short- and long-term EXOSC3 depletion conditions, which is especially evident for 3′ extended snoRNAs. The contribution of exonucleases to such decay is complex and its physiological relevance will require further analyses (see the ‘Discussion’ section).

## DISCUSSION

Most functional studies of nucleoplasmic RNA decay in mammalian cells have relied on siRNA-mediated factor depletion (18,19,26). The appearance of long-term side effects would not be all that surprising as RNA degradation is key to cellular homeostasis (3) and has been alluded to in studies focusing on exonucleases involved in nuclear RNA turnover (10,62). To directly interrogate the extent to which indirect effects have influenced previous RNAi-induced depletion experiments, we implemented the AID system for the rapid depletion of ZCCHC8, ZFC3H1 and EXOSC3, and combined this with global proteome analyses and the precise transcriptome-wide 3′ end mapping of newly synthesized RNA. Below, we discuss the major points arising from our analyses.

### The exosome and its cofactors mostly impact the proteome via their protein–protein interactions

Indirect effects of protein depletion may arise from ‘third-party’ contributions to the observed phenotype; for example, depletion of an interaction partner may compromise protein stability or render it more available for alternative interactions. While our experiments generally detected few proteomic changes, the temporal resolution achieved by the AID system served to group these into immediate or delayed responses to the respective factor depletion. For example, co-depletion events followed the depletion of target protein levels in a timely, consequential manner and were often found to be driven by the loss of a known interaction partner, as exemplified by the destabilization of RBM7 in ZCCHC8 depletions, which disarms the stable trimeric NEXT complex (70–72). Likewise, the stability of RNA exosome components may also be impacted by the interaction of individual subunits (66). In the present study, depletion of EXOSC3 induced the specific co-depletion of components cohabiting the exosome cap structure (EXOSC10, MPHOSPH6, C1D, EXOSC1), whereas no proteomic changes were observed for lower ring exosome subunits, including the directly connecting EXOSC5–EXOSC9 heterodimer (Figure 2E). This suggests a modular organization that discriminates exosome cap and ring moieties. We note that other studies have showed that long-term depletion of EXOSC5 negatively affects EXOSC3 levels (71,81), and that extended depletion of another cap component, EXOSC2, affects most subunits regardless of their location (66,82). Curiously, EXOSC2 was the only cap protein that was unaffected by our EXOSC3 depletion, implying that its stability depends on other interactions with the exosome core. Therefore, individual exosome components provide distinct contributions to complex integrity and acute subunit depletion may uncover proximity-linked co-dependence within submodules of the exosome core. These submodules will likely impact each other as indicated by the general destabilization of core ring subunits following long-term EXOSC3 depletion.

Long-term depletion of EXOSC3 also resulted in the decline of numerous ‘third-party’ proteins not directly related to exosome function. This more pronounced effect of EXOSC3 depletion, compared to those of ZCCHC8 and ZFC3H1, likely mirrors the broader function of the exosome in nuclear and cytoplasmic metabolism, as demonstrated by the broader transcriptomic phenotype and the co-depletion of the cytoplasmic SKI complex (see Figure 2F and Supplementary Figure S3A). Perhaps surprisingly, given the general upregulation of RNA, all of the depletion conditions resulted in only few upregulated proteins. We take this to suggest that the accumulating mRNAs remain nuclear retained (37) or that their increased cytoplasmic levels do not detectably impact translation at least within the time frame of our experiments.

In conclusion, we detected only sparse proteome deregulation in our experiments, especially upon ZCCHC8 and ZFC3H1 depletions, where short- and long-term factor depletion effects were largely unrecognizable. Yet, and as illustrated below, this does not ensure against indirect outcomes induced by long-term depletion.

### Rapid ZCCHC8 depletion demonstrates previous overestimation of NEXT’s contribution to the degradation of regular introns

At the transcriptomic level, rapid factor depletion generally enabled a refined definition of NEXT- and PAXT-sensitive 3′ ends. We were initially encouraged to recapitulate the cooperative targeting mode of NEXT and PAXT pathways, originally characterized through RNAi (26), but now revealed by rapid ZCCHC8 depletion and exacerbated accumulation of NEXT substrates in the co-depletion with ZFC3H1 (Figure 1C). Thus, the suggested substrate polyadenylation and handover to PAXT (25) is not solely the product of prolonged nuclear persistence of the target.

Other AID-defined targets displayed a relatively stronger adherence to expected exosome-sensitive substrates, such as PROMPTs and eRNAs, when comparing to previous target definitions based on RNAi (Figure 3G). This made us suspicious that the latter, particularly in the case of NEXT depletion, were confounded by the accumulation of indirect targets. In this regard, protein-coding regions exposed the most striking difference between short- and long-term depletion approaches, which was largely driven by the RNAi-specific accumulation of SA sites of regular introns (Figure 4A). This observation previously prompted a model that the NEXT-targeted exosome contributes to intron 3′–5′ exonucleolysis, supported by the general binding of RBM7 in the 3′ region of introns (24), the physical connection of NEXT with splicing factors (35) and an accumulation of snoRNA-hosting intron 3′ ends in exosome depletions (24,34). However, while our rapid depletion of ZCCHC8 confirmed NEXT targeting of 3′ extended intronic snoRNAs, regular introns failed to display a pronounced phenotype. Since degradation of excised and debranched introns can also be performed by 5′–3′ exonucleolytic decay (33,34), we interpret our results to suggest that 5′–3′ exonucleolysis is the primary intronic decay pathway and that NEXT-mediated degradation is only readily exposed with snoRNA-hosting introns where 5′–3′ exonucleolysis is blocked by intron-residing snoRNPs. That said, the increased NEXT sensitivity of longer introns, also detectable in the AID depletion data (Figure 4D), hints that bidirectional exonucleolysis may play a role for longer substrates. Here, the 3′–5′ exonucleolytic machinery may support 5′–3′ decay through its combinatorial exonucleolysis and/or aiding in resolving instances of stalled 5′–3′ exonucleases. Indeed, the latter may constitute a cause for general intron accumulation upon long-term NEXT depletion, where engaged 5′–3′ exonuclease may be overtly engaged/trapped on some substrates in the absence of 3′–5′ activity.

Overall, the ‘intron case’ exemplifies the range of phenotypes that are detected using different approaches for protein depletion. While short-term factor depletion enables mapping the primary consequences of immediate disrupted activities, long-term depletions are prone to be convoluted with indirect effects, complicating interpretation of target protein function. Nevertheless, long-term depletion approaches gain relevance when considering the physiological effects of factor absence, such as the indirect activation of signaling cascades involved in cellular defense pathways (Supplementary Figure S2A). As disease phenotypes often represent the consequences of long-term functional impairment, comparison here of short- and long-term depletion effects has the potential to distinguish ‘cause, consequence and/or compensation’, ultimately enabling a more comprehensive disease model.

### EXOSC3-independent 3′ end trimming disclosed

Although somewhat outside of the scope of the original aim of this study, our RNA 3′ end mapping data also uncovered core exosome-independent exonucleolysis of unprotected NEXT-sensitive 3′ ends (Figure 5A). This phenotype, most evident for 3′ extended and intron-hosted snoRNAs, was observed in both long- and short-term depletion conditions, filing it as an interesting direct consequence of EXOSC3 deficiency. As to which nucleases are responsible for this phenotype, we observed a rescue of trimming in the simultaneous absence of EXOSC3 with EXOSC10 or PARN. While this indicates that these 3′–5′ exonucleases might operate outside of context of the core exosome, the additional augmented 3′ end accumulation in co-depletion conditions suggests alternative targeting modes of EXOSC3 or PARN, which are stimulated in the absence of the primary RNA turnover pathway. Any physiological relevance of these activities requires further analysis, including a distinction between decay and processing with respect to which, possibly redundant, targeting mechanisms are involved. In the same vein, a previous study demonstrated unaltered levels of mature snoRNAs in the absence of EXOSC3 (24), suggesting complete intron degradation as a consequence of NEXT targeting. Furthermore, EXOSC10 and PARN have been linked to processing of short noncoding RNAs, but mostly in closer spatial proximity to the mature transcript (79). These studies, again, could benefit from the high-resolution kinetics of rapid depletion, combining co-depletions of nucleases in question to enable direct functional insights.

## DATA AVAILABILITY

The MS proteomics data have been deposited in the ProteomeXchange Consortium via the PRIDE partner repository with the dataset identifier PXD026774 and 10.6019/PXD026774. AID 3′ end sequencing data have been deposited in GEO under the accession number GSE179106.

RNAi 3′ end sequencing data were described in (26) and are available at GEO under accession code GSE137612. RNA sequencing data were described in (19,29) and are available at GEO under the accession codes GSE84172 and GSE151919, respectively. AID 3′ end sequencing data have been deposited in GEO under the accession number GSE179106.

## Supplementary Material

gkac001_Supplemental_Files

## Notes

Present address: Marvin Jens, Laboratory for Systems Biology of Gene Regulatory Elements, Berlin Institute for Medical Systems Biology, Max-Delbrück Center for Molecular Medicine, 10115 Berlin, Germany.
