# Supplementary material for: Rapid factor depletion highlights intricacies of nucleoplasmic RNA degradation
Source: Nucleic Acids Res. 2022 Jan 20;50(3):1583–600. doi: 10.1093/nar/gkac001 (PMC8860595; doi:10.1093/nar/gkac001)
Supplement: gkac001_Supplemental_Files [file gkac001_supplemental_files.zip › NAR_Supplement.pdf]

**Rapid factor depletion highlights intricacies of nucleoplasmic RNA degradation**

Maria Gockert[1], Manfred Schmid[1], Lis Jakobsen[2], Marvin Jens[3], Jens S. Andersen[2], Torben Heick Jensen[1,*]

# Supplemental figures and tables

**Supplemental Figure S1.** Related to Figure 1

**Supplemental Figure S2.** Related to Figure 2

**Supplemental Figure S3.** Related to Figure 3

**Supplemental Figure S4.** Related to Figure 4

**Supplemental Figure S5.** Related to Figure 5

**Supplemental Table S1.** sgRNA sequences used for CRISPR/Cas9-mediated KI. Related to experimental procedures

**Supplemental Table S2.** Primers used for genotyping PCR. Related to experimental procedures

**Supplemental Table S3.** siRNA sequences. Related to experimental procedures

**Supplemental Table S4.** Antibodies used for Western blotting. Related to experimental procedures

**Supplemental Table S5.** Primers used for RT-qPCR. Related to experimental procedures

**Supplemental Table S6.** Differential protein expression (DEP) results table. Related to Figure 2

**Figure S1.** A rapid depletion strategy provokes adaptor-specific substrate responses (related to Figure 1)
(**A**) Schematic representation of the CRISPR/Cas9-mediated knock-in (KI) strategy for endogenous mAID-tagging. Homology dependent repair was reliant on two donor cassettes harboring the mAID sequence, a T2A cleavage site and either hygromycin (HygroR) or neomycin (NeoR) resistance markers between the gene-specific homology arms (HA). Antibiotic double selection ensured correct insertion of the KI cassette at 2 alleles, thereby increasing the efficiency of homozygous mAID tagging.
(**B**) Genotyping PCR of genomic DNA isolated from unedited HeLa:TIR1 (ctrl), and monoclonal ZCCHC8 (left panel), ZFC3H1 (mid panel) and EXOSC3 (right panel) -mAID knock-in (KI) cells. Primers were designed to anneal outside of the homology arm sequence (indicated in A).
(**C**) RT-qPCR analysis of the indicated NEXT and PAXT targets from total RNA isolated from unedited HeLa:TIR1 (ctrl) cell lines upon 0h or 10h exposure to IAA. Results are shown relative to the 0h IAA sample and normalized to GAPDH mRNA. Display and statistical analysis as in Figure 1B.

**Figure S2:** Proteome effects upon factor depletions (related to Figure 2)
(**A**) GO-analysis based on the clustering from Figure 2C and showing the top 5 most significant GO-terms within each cluster. Absolute numbers of genes within a given GO-term are represented on the x-axis. Relative enrichment and significance are indicated by the color-scale and dot size.
(**B**) Clustered heatmap representation comparing changes in protein- and mRNA-levels upon factor depletions. Clustering and protein levels are as in Figure 2C, but only displaying the 28h timepoints (left columns). Changes in mRNA levels (right columns) were obtained from total RNA sequencing data in respective (ZCCHC8, ZFC3H1 and EXOSC3) siRNA KD conditions and calculated relative to a siEGFP control KD sample (Meola *et al.*, 2016). For each mAID-cell line, only proteins with significant log2FC after 6h or 28h of IAA-treatment (see Figure 2C) are displayed and ranked according to respective mRNA changes within each cluster. For RNA-seq data, transcripts with padj < 0.1 and log2FC relative to the control of < -1 or > 1 are indicated by black dots.
(**C**) Western blotting analysis of EXOSC3-mAID HeLa:TIR1 cells after exposure to 750 µM IAA for the indicated times periods. Blots were probed with endogenous antibodies against EXOSC3, EXOSC10, DIS3 and ZFC3H1. Actin (ACT) was used as a loading control.
(**D**) Genome browser view of selected genes showing total RNA sequencing data from siEXOSC3 and siEGFP (ctrl) KD conditions (Lykke-Andersen, Žumer, Molska, Rouvière, *et al.*, 2020). Respective gene annotations are displayed below each genome browser view.
(**E**) Western blotting analysis of unedited (ctrl) and ZCCHC8-mAID HeLa:TIR1 cells. Degron-tagged cell lines were exposed to 750 µM IAA for the indicated times periods). Blots were probed with endogenous antibodies against ZCCHC8, RBM7 and MTR4. Vinculin (VIN) was used as a loading control.
(**F**) Genome browser view of the RBM7 locus, displaying total RNA sequencing data from siZCCHC8 and siEGFP (ctrl) KD conditions (Meola *et al.*, 2016).

**Figure S3:** AID-mediated depletion enables identification of direct targets of NEXT and PAXT (related to Figure 3)
(**A**) Bar plots showing the numbers of significantly upregulated (padj < 0.1, log2FC > 1) $pA^{+}/pA^{+/-}$ 3'end clusters according to differential expression (DE) analyses upon AID- and RNAi-mediated depletion of ZCCHC8 and ZFC3H1. Numbers were calculated relative to the respective 0h IAA (mAID) and siEGFP (RNAi) control libraries (see schematic overview). Absolute values are indicated above each bar.

(**B**) RT-qPCR analysis of total RNA from ZCCHC8- and ZFC3H1-mAID cells treated with the respective siRNAs or with IAA for 6h. Values were normalized to RPO mRNA levels and plotted relative to results from respective 0h IAA (mAID) or siEGFP (RNAi) samples (see italics). Results are displayed as in Figure 1B.
(**C**) Western blotting analysis of cells from (B). Blots were probed with antibodies against endogenous ZCCHC8 or ZFC3H1 as well as Actin (ACT) as a loading control.
(**D**) Scatter plots comparing basal expression in $pA^{+/-}$ control libraries (siEGFP vs 0h IAA sample of ZCCHC8 and ZFC3H1-mAID). Displayed values represent log2 values of normalized read counts in 3'end clusters that exhibit significant upregulation upon 6h AID or RNAi mediated depletion of ZCCHC8 (left) or ZFC3H1(right). Orange lines mark equal expression between 2 conditions. Values centering around this orange line indicate equal basal expression in the displayed samples. Dashed lines represent log2 signal cutoffs, set to 2. Associated values on each side of the dashed lines indicate the numbers of 3'end clusters with signals lower and higher than 2, respectively.
(**E**) Boxplot representation of expression levels from (D). Mean log2 values are indicated below each bar.

**Figure S4: Rapid ZCCHC8-depletion establishes NEXT-targeting of snoRNA-hosting introns** (related to Figure 4)
(**A**) Quantification of metagene data from Figure 4D. Shown are log2FC of 3'end signals around SAs (4 nt up- and 2 nt down-stream) of regular introns, stratified according to their lengths (see Figure 4D) upon RNAi- or 6h AID-mediated depletion of ZCCHC8. Numbers shown below each box indicate the mean log2FC.
(**B**) Similar to Figure 4D, but displaying total RNA 3'end sequencing data from RNAi (G. Wu *et al.*, 2020).
(**C**) Similar to A, but containing signals from SA-sites of transcripts that do not host any snoRNAs (n=1000 per category).
(**D**) Similar to Figure 4E but showing SA sites of snoRNA hosting introns.
(**E**) Volcano plot displaying protein changes in ZCCHC8-mAID cells treated with IAA for 28h relative to the unedited ctrl (see Figure 2A). Displayed proteins are selected for being involved in splicing (GO:0000375, brown) or, more specifically, the post-mRNA release spliceosomal complex (GO:0071014, red) and in 5'-3'exonucleolysis (GO:0004534, blue). Significant proteins (padj < 0.05, see shape) are labeled. Log2FC threshold (compare Figure 2C) is indicated by dashed lines.

**Figure S5:** 3'end trimming in EXOSC3 depletion conditions (related to Figure 5)
(**A**) Heatmap representations of intron hosted snoRNAs, showing log2FC between $pA^{+}$ and $pA^{+/-}$ 3'end seq data of newly synthesized RNA upon RNAi or 6h AID-mediated ZCCHC8 (left) or EXOSC3 (right) depletion relative to respective controls (AID: HeLa:TIR1, 0h IAA and RNAi:siEGFP). Displayed region covers 500 bp upstream and 100 bp downstream of the SA. Left panel: All snoRNA introns, with dashed line indicating the 3'end of the snoRNA. Right panel: A selection of snoRNA introns that overlap annotated branchpoints (Briese *et al.*, 2019) with dashed line indicating the branchpoint position.
(**B**) Western blotting analysis of EXOSC3-mAID (upper panel) and ZCCHC8-mAID (lower panel) cells treated with siEGFP, siEXOSC10 or siPARN followed by 0h (-) or 6h (+) IAA exposure. Blots were probed with antibodies against endogenous ZCCHC8, EXOSC3, EXOSC10 and PARN. Actin (ACT) or tubulin (TUBULIN) were used as loading controls.
(**C**) RT-qPCR analysis displaying the individual values employed to calculate the ratio of distal/proximal amplicon values displayed in Figure 5C. Display and statistical analysis within amplicon-groups as in Figure 1B.

(**D**) Metagene profiles of regions around SA sites of long, regular introns (see Figure 4D, right panel), showing log2FC in single nucleotide resolution between $pA^{+}$ and $pA^{+/-}$ 3'end seq data of RNAi mediated ZCCHC8 and EXOSC3 depletion relative to siEGFP. Regions are displayed as in Figure 4D.

(**E**) Metagene profiles of data anchored around canonical 5' Drosha cleavage sites (Kim *et al.*, 2017) as schematisized. Values shown are log2FC in single nucleotide resolution between $pA^{+}$ and $pA^{+/-}$ 3'end-seq RNAi or 6h AID libraries following ZCCHC8 and EXOSC3 depletion relative to their respective controls. The displayed region covers 50 nt up- and 10 nt down-stream the cleavage site (anchor point).

**Supplemental Table S1.** sgRNA sequences used for CRISPR/Cas9-mediated KI. Related to experimental procedures

| Target gene | Sense orientation | Antisense orientation |
|---|---|---|
| EXOSC3 | CACCGTCAGTTGAGGCAAAAAACTA | AAACTAGTTTTTTGCCTCAACTGAC |
| ZCCHC8 | CACCGTAAGTCAAGCCATTATTCAG | AAACCTGAATAATGGCTTGACTTAC |
| ZFC3H1 | CACCGAAGCAAGAATCACTGAACAC | AAACGTGTTCAGTGATTCTTGCTTC |

**Supplemental Table S2.** Primers used for genotyping PCR. Related to experimental procedures

| Target gene | Sense orientation | Antisense orientation |
|---|---|---|
| EXOSC3 | AGCGTGGGTGAAAACCAGTA | ATTATGGGGGTCACACGCAG |
| ZCCHC8 | CCATCACTCTCGACGGATAGTC | GGATGAGGACGCACTGACTC |
| ZFC3H1 | GGCAAGGAGTCCAGAACGTAG | CCTCCTTGGAAGTTTAGCCTG |

**Supplemental Table S3.** siRNA sequences. Related to experimental procedures

| Target gene | Sense orientation | Antisense orientation |
|---|---|---|
| EGFP | GACGUAAACGGCCACAAGUdTdT | ACUUGUGGCCGUUUACGUCdTdT |
| ZCCHC8 | GGAAUGUACCUCAGGAUAAdTdT | UUAUCCUGAGGUACAUUCCdTdT |
| ZFC3H1 | GAUUAGAGUCCAUGAUUAAdTdT | UUAAUCAUGGACUCUAAUCdTdT |
| EXOSC10 | CCAGUUAUACAGACCUAUAdTdT | UAUAGGUCUGUAUAACUGGdTdT |
| PARN | AGGCAUUCAUGUUGAGACUdTdT | AGUCUCAACAUGAAUGCCUdTdT |

**Supplemental Table S4.** Antibodies used for Western blotting. Related to experimental procedures

| name | Distributer, reference number | host | dilution |
|---|---|---|---|
| EXOSC3 | Nordic Biosite, 15062-1-AP | rabbit | 1:1000 |
| MTR4 | Abcam, ab70551 | rabbit | 1:2000 |
| ZCCHC8 | Novus Biologicals, NB100-94955 | rabbit | 1:1000 |
| ZFC3H1 | Sigma, HPA-00715 | rabbit | 1:1000 |
| Alpha-Tubulin (TUB) | Rockland, 200-301-880 | rabbit | 1:2500 |
| Beta-Actin (ACT) | Sigma, A2228 | mouse | 1:10000 |
| DIS3 | Sigma, HPA039281 | rabbit | 1:1000 |
| Vinculin (VIN) | Sigma-Aldrich, V9131 | mouse | 1:500000 |
| EXOSC10 | Santa Cruz, sc-374595 | mouse | 1:1000 |
| RBM7 | Proteintech, 21896-1-AP | rabbit | 1:1000 |
| PARN | Abcam, ab188333 | rabbit | 1:1000 |

**Supplemental Table S5.** Primers used for RT-qPCR. Related to experimental procedures

| Target | Sense orientation | Antisense orientation |
|---|---|---|
| proMGST3 | GCAGTCTCCGGTGTAAAATGA | AGTCATTCCAAGGTGGTGGA |
| proDNAJB4 | TTTCTGGCGTTTCTGATTGA | ACCAAAACGCAGGTTGTTTA |
| proPSMC3IP | TTTCGAGACCCAGTTCCAGC | GTCCCTGTGGGGTAACCTTG |

| | | |
|---|---|---|
| SNHG19 | CGTCCAGGCCTGGCCTAC | GCTCGCGACGAAACCTGC |
| proSAMD4B | CTTAGCGCTTTCTGTGCATTCT | CTCTCCGATGGGAATGGCGA |
| proLMO4 | AGGCTAGCACCCTAAAAGCA | CTTTCCCGACTACCCTCCTC |
| proKLF6 | AAGTTTTAGAGGGTCCGGCA | CTCTGCATAACCTTCACCG |
| proRBM39 | AATAGATTTCCCTGTCATTTGGAGC | TTTCCAAGGTTGTTTCAAAGCTCG |
| proSTAT3 | CCGCCTGCTTTGAACTTCAG | TCTCCCCACGCACTCTAGTA |
| proTTC32 | GTCTGTTCCACGGTCCAAAC | ACAGCAGGCATGTAGGGTAG |
| proASH1L | TAGGGAGTGAGGCCAGTAGGA | TCCCAGGTTGGCAACTCAAC |
| proDDX6 | CACACCGACGAGAAAAGTTCG | CATTTCTCAATCACGTCGCGG |
| SNORD91A_prox | GTTTGCCTTCTTTCAGTTTAGGCA | GGGGACCAAGTAAGAAAAACAAA |
| SNORD91A_dist | TTGGTGGCCAGTAGTTGTGT | CCACACTCATTCAAGCCCCT |
| SNORA46_prox | ATAGCACTGTTGAATCATAAACACA | AAGAACAGGCCCCAAAGTAGA |
| SNORA46_dist | TGCTTGCTGCCTTTAAGGACT | TATGTGCCAGCTATCCTCCC |
| SNORD83A_prox | AGGGGAGACCTGTGGGTAAT | TGACCCCTTCCTGCTACTCA |
| SNORD83A_dist | TCCTGGACCCTTTGCTCTTTG | TTAAGCTCCCCCATTCACAG |
| RPO | TCCATTGTGGGAGCAGAC | CAGCAGTTTCTCCCAGAGC |
| GAPDH | GTCAGCCGCATCTTCTT | GCGCCCAATACGACCAAATC |

**Supplemental Table S6.** DEP-result table, arranged according to ascending log2FC within the individual tagged cell lines (EXOSC3-, ZCCHC8-, ZFC3H1-mAID). Related to Figure 2.

| name | ID | Tagged protein | depletion | log2FC | padj | pval |
|---|---|---|---|---|---|---|
| EXOSC3 | Q9NQT5 | EXOSC3 | 6h | -3,63 | 1,33E-10 | 8,9688E-14 |
| CPA4 | Q9UI42 | EXOSC3 | 28h | -2,63 | 0,00046 | 2,1038E-16 |
| SLC1A3 | P43003 | EXOSC3 | 28h | -2,35 | 0,00633 | 5,7107E-15 |
| ALDH1B1 | P30837 | EXOSC3 | 6h | -2,29 | 4,28E-14 | 6,5347E-17 |
| LRRC58 | Q96CX6 | EXOSC3 | 28h | -2,29 | 0,000399 | 1,8262E-16 |
| ALDH1B1 | P30837 | EXOSC3 | 28h | -2,27 | 0,000176 | 7,7698E-17 |
| ALDH1B1 | P30837 | EXOSC3 | 0h | -2,15 | 4,14E-14 | 2,3312E-16 |
| TCAF1 | Q9Y4C2 | EXOSC3 | 28h | -1,99 | 0,0192 | 3,7887E-14 |
| APOB | P04114 | EXOSC3 | 28h | -1,97 | 7,81E-07 | 1,2448E-18 |
| LRRC58 | Q96CX6 | EXOSC3 | 6h | -1,96 | 4,28E-14 | 3,9478E-15 |
| SERPINB5 | P36952 | EXOSC3 | 28h | -1,93 | 1,68E-13 | 5,9124E-24 |
| SERPINB5 | P36952 | EXOSC3 | 0h | -1,91 | 4,14E-14 | 7,6514E-24 |
| EXOSC1 | Q9Y3B2 | EXOSC3 | 28h | -1,9 | 0,0000158 | 1,0231E-17 |
| SERPINB5 | P36952 | EXOSC3 | 6h | -1,89 | 4,28E-14 | 9,2448E-24 |
| LRRC58 | Q96CX6 | EXOSC3 | 0h | -1,89 | 4,14E-14 | 7,7137E-15 |
| PDCD4 | Q53EL6 | EXOSC3 | 28h | -1,89 | 1,68E-13 | 4,5421E-22 |
| JAK1 | P23458 | EXOSC3 | 28h | -1,85 | 1,03E-07 | 3,5798E-19 |
| LGALS3BP | Q08380 | EXOSC3 | 28h | -1,85 | 6,46E-10 | 3,1795E-20 |
| SLC1A3 | P43003 | EXOSC3 | 6h | -1,84 | 1,01E-08 | 5,9676E-13 |
| TTC37 | Q6PGP7 | EXOSC3 | 28h | -1,82 | 1,24E-07 | 3,9832E-19 |
| MRFAP1 | Q9Y605 | EXOSC3 | 28h | -1,8 | 0,0156 | 2,6296E-14 |
| MYO10 | Q9HD67 | EXOSC3 | 28h | -1,8 | 2,62E-07 | 6,3777E-19 |
| SKIV2L | Q15477 | EXOSC3 | 28h | -1,8 | 1,68E-13 | 4,5843E-22 |
| SQSTM1 | Q13501 | EXOSC3 | 28h | -1,79 | 5,86E-11 | 1,219E-20 |

| | | | | | | |
|---|---|---|---|---|---|---|
| IL6ST | P40189 | EXOSC3 | 28h | -1,75 | 0,0000687 | 3,297E-17 |
| SLC1A3 | P43003 | EXOSC3 | 0h | -1,74 | 2,78E-08 | 1,7563E-12 |
| MTARC1 | Q5VT66 | EXOSC3 | 28h | -1,68 | 0,00000534 | 4,6954E-18 |
| MTDH | Q86UE4 | EXOSC3 | 28h | -1,68 | 0,00000382 | 3,5856E-18 |
| CPA4 | Q9UI42 | EXOSC3 | 6h | -1,66 | 5,31E-08 | 1,3962E-12 |
| KLHL13 | Q9P2N7 | EXOSC3 | 28h | -1,61 | 0,0000978 | 4,5339E-17 |
| RRBP1 | Q9P2E9 | EXOSC3 | 28h | -1,6 | 1,68E-13 | 8,4387E-22 |
| CPA4 | Q9UI42 | EXOSC3 | 0h | -1,58 | 9,1E-08 | 3,3047E-12 |
| MORF4L1 | Q9UBU8 | EXOSC3 | 28h | -1,58 | 0,00000223 | 2,4528E-18 |
| TSPAN3 | O60637 | EXOSC3 | 28h | -1,52 | 0,0237 | 5,6468E-14 |
| PRSS56 | P0CW18 | EXOSC3 | 28h | -1,51 | 0,00000619 | 5,2905E-18 |
| DDR1 | Q08345 | EXOSC3 | 28h | -1,48 | 0,000109 | 5,0205E-17 |
| LAMC1 | P11047 | EXOSC3 | 28h | -1,45 | 5,75E-07 | 1,0223E-18 |
| FDFT1 | P37268 | EXOSC3 | 28h | -1,44 | 0,000287 | 1,2751E-16 |
| WLS | Q5T9L3 | EXOSC3 | 28h | -1,44 | 0,0000054 | 4,7409E-18 |
| EXOSC3 | Q9NQT5 | EXOSC3 | 0h | -1,41 | 0,0434 | 1,0747E-06 |
| DHFR | P00374 | EXOSC3 | 28h | -1,41 | 1,16E-09 | 4,254E-20 |
| HMGCS1 | Q01581 | EXOSC3 | 28h | -1,39 | 0,0000446 | 2,2677E-17 |
| STAU1 | O95793 | EXOSC3 | 28h | -1,39 | 0,028 | 7,8591E-14 |
| TIMMDC1 | Q9NPL8 | EXOSC3 | 28h | -1,39 | 0,000133 | 5,9742E-17 |
| TSPYL1 | Q9H0U9 | EXOSC3 | 28h | -1,39 | 0,001 | 5,0163E-16 |
| CDKN2A | P42771 | EXOSC3 | 28h | -1,38 | 0,0000036 | 3,4293E-18 |
| FNDC3B | Q53EP0 | EXOSC3 | 28h | -1,38 | 0,03 | 9,0695E-14 |
| TIMELESS | Q9UNS1 | EXOSC3 | 28h | -1,35 | 0,00000479 | 4,2859E-18 |
| EXOSC10 | Q01780 | EXOSC3 | 6h | -1,31 | 0,00303 | 8,8621E-09 |
| CTNNA1 | P35221 | EXOSC3 | 28h | -1,31 | 3,69E-09 | 7,1251E-20 |
| POLR2A | P24928 | EXOSC3 | 28h | -1,3 | 4,4E-09 | 7,7698E-20 |
| LAMC1 | P11047 | EXOSC3 | 6h | -1,29 | 4,28E-14 | 9,6672E-18 |
| TNS3 | Q68CZ2 | EXOSC3 | 28h | -1,27 | 6,32E-07 | 1,0859E-18 |
| UBE2C | O00762 | EXOSC3 | 28h | -1,27 | 0,00000372 | 3,5179E-18 |
| KIAA1671 | Q9BY89 | EXOSC3 | 28h | -1,26 | 0,00119 | 6,301E-16 |
| CRABP2 | P29373 | EXOSC3 | 6h | -1,25 | 0,00000125 | 8,8025E-12 |
| HELLS | Q9NRZ9 | EXOSC3 | 28h | -1,25 | 1,04E-12 | 3,1454E-21 |
| NOB1 | Q9ULX3 | EXOSC3 | 28h | -1,24 | 0,00718 | 6,9264E-15 |
| UBE2T | Q9NPD8 | EXOSC3 | 28h | -1,24 | 0,000314 | 1,4121E-16 |
| MTR | Q99707 | EXOSC3 | 28h | -1,23 | 1,82E-07 | 5,0598E-19 |
| OAS1 | P00973 | EXOSC3 | 0h | -1,22 | 0,0211 | 2,6437E-07 |
| DLGAP5 | Q15398 | EXOSC3 | 28h | -1,22 | 0,0000762 | 3,6282E-17 |
| CRABP2 | P29373 | EXOSC3 | 0h | -1,21 | 0,00000143 | 1,6943E-11 |
| LAMC1 | P11047 | EXOSC3 | 0h | -1,2 | 4,14E-14 | 4,2961E-17 |
| L1CAM | P32004 | EXOSC3 | 28h | -1,2 | 0,00104 | 5,2307E-16 |
| ACBD6 | Q9BR61 | EXOSC3 | 28h | -1,19 | 0,02 | 4,0569E-14 |
| CDC123 | O75794 | EXOSC3 | 28h | -1,19 | 5,43E-10 | 2,9326E-20 |
| CYP51A1 | Q16850 | EXOSC3 | 28h | -1,19 | 3,15E-08 | 1,9122E-19 |
| EHD2 | Q9NZN4 | EXOSC3 | 28h | -1,17 | 5,13E-08 | 2,4618E-19 |

| | | | | | | |
|---|---|---|---|---|---|---|
| RRM2 | P31350 | EXOSC3 | 28h | -1,16 | 0,00000131 | 1,7085E-18 |
| ITGA2 | P17301 | EXOSC3 | 0h | -1,13 | 0,0000724 | 2,9247E-10 |
| FAM65A | Q6ZS17 | EXOSC3 | 28h | -1,12 | 0,000103 | 4,7424E-17 |
| TRIM16 | O95361 | EXOSC3 | 28h | -1,12 | 0,00138 | 7,7497E-16 |
| ITGA2 | P17301 | EXOSC3 | 6h | -1,11 | 0,000186 | 3,9063E-10 |
| UHRF1 | Q96T88 | EXOSC3 | 28h | -1,11 | 0,00000493 | 4,3946E-18 |
| CTNNB1 | P35222 | EXOSC3 | 28h | -1,1 | 0,00948 | 1,096E-14 |
| SND1 | Q7KZF4 | EXOSC3 | 28h | -1,1 | 1,68E-13 | 1,73E-21 |
| CTNNAL1 | Q9UBT7 | EXOSC3 | 28h | -1,09 | 0,00856 | 9,2029E-15 |
| FAM83H | Q6ZRV2 | EXOSC3 | 28h | -1,09 | 0,00253 | 1,6991E-15 |
| ITGA3 | P26006 | EXOSC3 | 0h | -1,08 | 0,000747 | 2,8043E-09 |
| TIMM23 | O14925 | EXOSC3 | 28h | -1,07 | 0,00269 | 1,8393E-15 |
| CBR3 | O75828 | EXOSC3 | 6h | -1,06 | 0,000865 | 1,9582E-09 |
| ERMP1 | Q7Z2K6 | EXOSC3 | 6h | -1,06 | 4,28E-14 | 1,2212E-18 |
| ABCG2 | Q9UNQ0 | EXOSC3 | 6h | -1,05 | 4,28E-14 | 3,3686E-16 |
| MYO10 | Q9HD67 | EXOSC3 | 6h | -1,05 | 3,67E-12 | 2,4475E-14 |
| CDK4 | P11802 | EXOSC3 | 28h | -1,05 | 0,000222 | 9,7599E-17 |
| KIFC3 | Q9BVG8 | EXOSC3 | 28h | -1,05 | 0,00108 | 5,4908E-16 |
| NDC80 | O14777 | EXOSC3 | 28h | -1,05 | 0,0323 | 1,0536E-13 |
| ERMP1 | Q7Z2K6 | EXOSC3 | 0h | -1,04 | 4,14E-14 | 1,8176E-18 |
| IMPDH1 | P20839 | EXOSC3 | 6h | -1,03 | 4,28E-14 | 4,9681E-15 |
| ITGA3 | P26006 | EXOSC3 | 6h | -1,03 | 0,00246 | 6,8302E-09 |
| AKAP9 | Q99996 | EXOSC3 | 28h | -1,03 | 8,58E-10 | 3,662E-20 |
| CASP2 | P42575 | EXOSC3 | 28h | -1,03 | 0,0000772 | 3,6701E-17 |
| TTC37 | Q6PGP7 | EXOSC3 | 6h | -1,02 | 7,4E-12 | 3,1382E-14 |
| FAM3C | Q92520 | EXOSC3 | 28h | -1,02 | 0,00903 | 1,0064E-14 |
| LAMB1 | P07942 | EXOSC3 | 6h | -1,01 | 4,28E-14 | 1,2847E-15 |
| ABCG2 | Q9UNQ0 | EXOSC3 | 0h | -1,01 | 4,14E-14 | 6,9556E-16 |
| ISG20L2 | Q9H9L3 | EXOSC3 | 28h | -1,01 | 0,0264 | 6,998E-14 |
| FYTTD1 | Q96QD9 | EXOSC3 | 6h | 1,01 | 0,0000154 | 5,0666E-11 |
| H1F0 | P07305 | EXOSC3 | 6h | 1,03 | 0,00000412 | 1,9309E-11 |
| TRIM29 | Q14134 | EXOSC3 | 6h | 1,04 | 4,28E-14 | 1,1652E-16 |
| RAB11FIP1 | Q6WKZ4 | EXOSC3 | 0h | 1,06 | 9,14E-12 | 5,9932E-14 |
| TFRC | P02786 | EXOSC3 | 28h | 1,1 | 0,00231 | 1,5067E-15 |
| PALM | O75781 | EXOSC3 | 6h | 1,11 | 0,00327 | 9,759E-09 |
| TRIM29 | Q14134 | EXOSC3 | 0h | 1,11 | 4,14E-14 | 3,1267E-17 |
| OASL | Q15646 | EXOSC3 | 6h | 1,12 | 4,28E-14 | 5,3087E-16 |
| CHCHD6 | Q9BRQ6 | EXOSC3 | 28h | 1,16 | 0,0475 | 2,4451E-13 |
| HPDL | Q96IR7 | EXOSC3 | 28h | 1,2 | 0,00000652 | 5,5189E-18 |
| KCTD12 | Q96CX2 | EXOSC3 | 28h | 1,21 | 0,00739 | 7,2403E-15 |
| LETMD1 | Q6P1Q0 | EXOSC3 | 28h | 1,21 | 0,00000194 | 2,2225E-18 |
| ISG15 | P05161 | EXOSC3 | 6h | 1,22 | 1,43E-07 | 2,4046E-12 |
| PALM | O75781 | EXOSC3 | 0h | 1,23 | 0,000493 | 1,7804E-09 |
| SLC12A7 | Q9Y666 | EXOSC3 | 0h | 1,24 | 4,14E-14 | 1,9E-17 |
| NCBP2- | Q69YL0 | EXOSC3 | 6h | 1,25 | 5,15E-13 | 1,2798E-14 |

| | | | | | | |
|---|---|---|---|---|---|---|
| AS2 | | | | | | |
| UNC13D | Q70J99 | EXOSC3 | 6h | 1,26 | 4,28E-14 | 6,5891E-20 |
| UNC13D | Q70J99 | EXOSC3 | 0h | 1,28 | 4,14E-14 | 5,3297E-20 |
| ISG15 | P05161 | EXOSC3 | 0h | 1,29 | 6,14E-09 | 8,3119E-13 |
| OASL | Q15646 | EXOSC3 | 0h | 1,29 | 4,14E-14 | 3,3083E-17 |
| ZFC3H1 | O60293 | EXOSC3 | 28h | 1,3 | 0,00000192 | 2,2034E-18 |
| SLC12A7 | Q9Y666 | EXOSC3 | 6h | 1,31 | 4,28E-14 | 6,1319E-18 |
| NES | P48681 | EXOSC3 | 28h | 1,32 | 3,36E-07 | 7,417E-19 |
| SCCPDH | Q8NBX0 | EXOSC3 | 6h | 1,33 | 2,45E-07 | 3,274E-12 |
| DUSP9 | Q99956 | EXOSC3 | 6h | 1,34 | 0,0248 | 2,1445E-07 |
| DUSP9 | Q99956 | EXOSC3 | 0h | 1,34 | 0,0185 | 2,0996E-07 |
| NES | P48681 | EXOSC3 | 0h | 1,37 | 4,14E-14 | 3,3012E-19 |
| ALPP | P05187 | EXOSC3 | 28h | 1,37 | 0,000672 | 3,136E-16 |
| PTBP2 | Q9UKA9 | EXOSC3 | 28h | 1,37 | 0,000202 | 8,8843E-17 |
| NES | P48681 | EXOSC3 | 6h | 1,38 | 4,28E-14 | 3,2018E-19 |
| ALPP | P05187 | EXOSC3 | 6h | 1,4 | 4,28E-14 | 1,9812E-16 |
| SCCPDH | Q8NBX0 | EXOSC3 | 0h | 1,41 | 1,03E-08 | 1,0824E-12 |
| ISG15 | P05161 | EXOSC3 | 28h | 1,44 | 0,0319 | 1,0263E-13 |
| SLC12A7 | Q9Y666 | EXOSC3 | 28h | 1,5 | 1,59E-07 | 4,6302E-19 |
| ALPP | P05187 | EXOSC3 | 0h | 1,51 | 4,14E-14 | 4,8204E-17 |
| SPRYD4 | Q8WW59 | EXOSC3 | 28h | 1,53 | 0,00164 | 9,8601E-16 |
| NCBP2-AS2 | Q69YL0 | EXOSC3 | 28h | 1,7 | 0,000071 | 3,3956E-17 |
| SCCPDH | Q8NBX0 | EXOSC3 | 28h | 1,76 | 0,0118 | 1,6116E-14 |
| SORBS2 | O94875 | EXOSC3 | 0h | 1,8 | 4,14E-14 | 3,4502E-24 |
| SORBS2 | O94875 | EXOSC3 | 6h | 1,81 | 4,28E-14 | 3,1974E-24 |
| H1F0 | P07305 | EXOSC3 | 28h | 1,81 | 0,000832 | 4,0012E-16 |
| SORBS2 | O94875 | EXOSC3 | 28h | 1,92 | 1,68E-13 | 9,5121E-25 |
| ACTL8 | Q9H568 | EXOSC3 | 28h | 2,18 | 1,68E-13 | 1,2153E-26 |
| ACTL8 | Q9H568 | EXOSC3 | 6h | 2,44 | 4,28E-14 | 1,2944E-27 |
| ACTL8 | Q9H568 | EXOSC3 | 0h | 2,45 | 4,14E-14 | 1,1728E-27 |
| ANXA1 | P04083 | EXOSC3 | 0h | 3,56 | 4,14E-14 | 1,7604E-26 |
| ANXA1 | P04083 | EXOSC3 | 6h | 3,58 | 4,28E-14 | 1,5727E-26 |
| ACAT2 | Q9BWD1 | EXOSC3 | 6h | 3,88 | 4,28E-14 | 1,3105E-16 |
| ACAT2 | Q9BWD1 | EXOSC3 | 28h | 3,94 | 0,000219 | 9,6078E-17 |
| ANXA1 | P04083 | EXOSC3 | 28h | 3,96 | 1,68E-13 | 2,028E-27 |
| EPB41L3 | Q9Y2J2 | EXOSC3 | 6h | 4 | 0,0000236 | 6,8859E-11 |
| EPB41L3 | Q9Y2J2 | EXOSC3 | 0h | 4,01 | 0,0000109 | 6,652E-11 |
| ACAT2 | Q9BWD1 | EXOSC3 | 0h | 4,05 | 4,14E-14 | 5,594E-17 |
| ZCCHC8 | Q6NZY4 | ZCCHC8 | 6h | -3,18 | 6,96E-14 | 6,4165E-17 |
| ZCCHC8 | Q6NZY4 | ZCCHC8 | 28h | -2,41 | 1,8E-09 | 1,3511E-14 |
| ABCB1 | P08183 | ZCCHC8 | 0h | -1,85 | 0,0000198 | 8,3949E-12 |
| CRABP2 | P29373 | ZCCHC8 | 0h | -1,66 | 2,33E-09 | 4,2602E-14 |
| SULT1A1 | P50225 | ZCCHC8 | 28h | -1,62 | 5,71E-14 | 7,6956E-20 |
| CRABP2 | P29373 | ZCCHC8 | 28h | -1,61 | 6,29E-08 | 7,5904E-14 |

| | | | | | | |
|---|---|---|---|---|---|---|
| CYP1B1 | Q16678 | ZCCHC8 | 0h | -1,6 | 0,000667 | 2,3656E-10 |
| SULT1A1 | P50225 | ZCCHC8 | 0h | -1,59 | 6,59E-14 | 1,2332E-19 |
| CRABP2 | P29373 | ZCCHC8 | 6h | -1,52 | 4,47E-08 | 2,2466E-13 |
| SULT1A1 | P50225 | ZCCHC8 | 6h | -1,5 | 6,96E-14 | 3,5511E-19 |
| TACC1 | O75410 | ZCCHC8 | 0h | -1,47 | 0,000137 | 4,8109E-11 |
| FDFT1 | P37268 | ZCCHC8 | 28h | -1,46 | 5,71E-14 | 1,0104E-16 |
| NDRG1 | Q92597 | ZCCHC8 | 28h | -1,44 | 1,28E-08 | 3,3847E-14 |
| ABCB1 | P08183 | ZCCHC8 | 6h | -1,42 | 0,00184 | 9,4266E-10 |
| HMGCS1 | Q01581 | ZCCHC8 | 28h | -1,42 | 5,71E-14 | 1,5628E-17 |
| HMGCS1 | Q01581 | ZCCHC8 | 6h | -1,35 | 6,96E-14 | 4,2172E-17 |
| HMGCS1 | Q01581 | ZCCHC8 | 0h | -1,35 | 6,59E-14 | 4,4788E-17 |
| ALDH1B1 | P30837 | ZCCHC8 | 28h | -1,33 | 0,0000115 | 2,1608E-12 |
| FDFT1 | P37268 | ZCCHC8 | 0h | -1,32 | 6,59E-14 | 6,8611E-16 |
| TACC1 | O75410 | ZCCHC8 | 6h | -1,31 | 0,000821 | 3,6003E-10 |
| C5orf22 | Q49AR2 | ZCCHC8 | 28h | -1,28 | 0,00000402 | 1,0225E-12 |
| SERPINB5 | P36952 | ZCCHC8 | 28h | -1,27 | 5,71E-14 | 2,7519E-20 |
| FDFT1 | P37268 | ZCCHC8 | 6h | -1,25 | 3,13E-13 | 1,8615E-15 |
| SLC2A3 | P11169 | ZCCHC8 | 28h | -1,22 | 0,00303 | 5,9245E-10 |
| LAMA1 | P25391 | ZCCHC8 | 28h | -1,21 | 0,0467 | 7,2262E-08 |
| ALDH1B1 | P30837 | ZCCHC8 | 6h | -1,2 | 0,0000291 | 1,5057E-11 |
| C5orf22 | Q49AR2 | ZCCHC8 | 6h | -1,2 | 0,00000424 | 3,4486E-12 |
| SLC2A3 | P11169 | ZCCHC8 | 0h | -1,17 | 0,00267 | 1,3252E-09 |
| NDRG1 | Q92597 | ZCCHC8 | 0h | -1,16 | 0,00000249 | 1,8791E-12 |
| SERPINB5 | P36952 | ZCCHC8 | 6h | -1,15 | 6,96E-14 | 1,7897E-19 |
| EML2 | O95834 | ZCCHC8 | 28h | -1,15 | 3,66E-11 | 2,7773E-15 |
| C5orf22 | Q49AR2 | ZCCHC8 | 0h | -1,14 | 0,0000233 | 9,5826E-12 |
| SERPINB5 | P36952 | ZCCHC8 | 0h | -1,13 | 6,59E-14 | 2,792E-19 |
| CTSZ | Q9UBR2 | ZCCHC8 | 28h | -1,13 | 0,0136 | 5,9352E-09 |
| RPL22L1 | Q6P5R6 | ZCCHC8 | 28h | -1,13 | 4,61E-08 | 6,4329E-14 |
| LAMA1 | P25391 | ZCCHC8 | 6h | -1,12 | 0,0473 | 2,3159E-07 |
| SLC2A3 | P11169 | ZCCHC8 | 6h | -1,12 | 0,0038 | 2,6202E-09 |
| RANGRF | Q9HD47 | ZCCHC8 | 28h | -1,12 | 0,00166 | 2,7074E-10 |
| ZCCHC8 | Q6NZY4 | ZCCHC8 | 0h | -1,11 | 0,0142 | 1,6477E-08 |
| CTSZ | Q9UBR2 | ZCCHC8 | 0h | -1,1 | 0,0101 | 9,1589E-09 |
| STAU1 | O95793 | ZCCHC8 | 28h | -1,09 | 0,0000543 | 7,5142E-12 |
| NDRG1 | Q92597 | ZCCHC8 | 6h | -1,07 | 0,000016 | 9,322E-12 |
| TACC1 | O75410 | ZCCHC8 | 28h | -1,06 | 0,0211 | 1,3256E-08 |
| ALDH1B1 | P30837 | ZCCHC8 | 0h | -1,05 | 0,000503 | 1,7276E-10 |
| RANGRF | Q9HD47 | ZCCHC8 | 0h | -1,03 | 0,00251 | 1,2135E-09 |
| EML2 | O95834 | ZCCHC8 | 6h | -1,02 | 3,64E-10 | 2,5863E-14 |
| RBPMS | Q93062 | ZCCHC8 | 28h | -1,02 | 0,0254 | 1,9286E-08 |
| SLC1A3 | P43003 | ZCCHC8 | 28h | -1,01 | 0,0295 | 2,6204E-08 |
| LEPREL1 | Q8IVL5 | ZCCHC8 | 6h | 1,01 | 0,00539 | 4,3624E-09 |
| ECHDC3 | Q96DC8 | ZCCHC8 | 0h | 1,01 | 6,59E-14 | 1,1233E-16 |
| LEPREL1 | Q8IVL5 | ZCCHC8 | 0h | 1,03 | 0,00523 | 3,3662E-09 |

| | | | | | | |
|---|---|---|---|---|---|---|
| ZNF768 | Q9H5H4 | ZCCHC8 | 28h | 1,04 | 0,0194 | 1,1226E-08 |
| IFIT1 | P09914 | ZCCHC8 | 28h | 1,06 | 0,0000149 | 2,6593E-12 |
| H1F0 | P07305 | ZCCHC8 | 0h | 1,07 | 0,0000246 | 1,004E-11 |
| OPA1 | O60313 | ZCCHC8 | 6h | 1,08 | 6,96E-14 | 3,3198E-17 |
| NCBP2-AS2 | Q69YL0 | ZCCHC8 | 0h | 1,08 | 7,23E-08 | 2,2673E-13 |
| OPA1 | O60313 | ZCCHC8 | 28h | 1,08 | 5,71E-14 | 3,3928E-17 |
| NCBP2-AS2 | Q69YL0 | ZCCHC8 | 6h | 1,09 | 2,59E-08 | 1,7204E-13 |
| OPA1 | O60313 | ZCCHC8 | 0h | 1,09 | 6,59E-14 | 3,0335E-17 |
| H1F0 | P07305 | ZCCHC8 | 6h | 1,1 | 0,00000834 | 5,696E-12 |
| HPDL | Q96IR7 | ZCCHC8 | 28h | 1,12 | 5,71E-14 | 2,3493E-17 |
| RCN3 | Q96D15 | ZCCHC8 | 6h | 1,14 | 5,94E-11 | 1,2186E-14 |
| RCN3 | Q96D15 | ZCCHC8 | 0h | 1,14 | 1,3E-10 | 1,2689E-14 |
| RCN3 | Q96D15 | ZCCHC8 | 28h | 1,14 | 1,32E-09 | 1,172E-14 |
| SCCPDH | Q8NBX0 | ZCCHC8 | 28h | 1,15 | 0,000346 | 4,3421E-11 |
| SLC12A7 | Q9Y666 | ZCCHC8 | 0h | 1,16 | 6,59E-14 | 7,0123E-17 |
| SLC12A7 | Q9Y666 | ZCCHC8 | 28h | 1,16 | 5,71E-14 | 7,8529E-17 |
| IFIT1 | P09914 | ZCCHC8 | 0h | 1,17 | 1,79E-07 | 3,7488E-13 |
| SLC12A7 | Q9Y666 | ZCCHC8 | 6h | 1,19 | 6,96E-14 | 4,2069E-17 |
| SCCPDH | Q8NBX0 | ZCCHC8 | 0h | 1,19 | 0,0000711 | 2,6357E-11 |
| SCCPDH | Q8NBX0 | ZCCHC8 | 6h | 1,22 | 0,0000281 | 1,4662E-11 |
| NES | P48681 | ZCCHC8 | 0h | 1,29 | 6,59E-14 | 1,0875E-18 |
| NES | P48681 | ZCCHC8 | 6h | 1,35 | 6,96E-14 | 4,625E-19 |
| SORBS2 | O94875 | ZCCHC8 | 28h | 1,36 | 5,71E-14 | 1,045E-21 |
| SORBS2 | O94875 | ZCCHC8 | 0h | 1,42 | 6,59E-14 | 3,97E-22 |
| SORBS2 | O94875 | ZCCHC8 | 6h | 1,45 | 6,96E-14 | 2,93E-22 |
| NCBP2-AS2 | Q69YL0 | ZCCHC8 | 28h | 1,51 | 5,71E-14 | 3,499E-16 |
| NES | P48681 | ZCCHC8 | 28h | 1,51 | 5,71E-14 | 5,2572E-20 |
| OASL | Q15646 | ZCCHC8 | 6h | 1,53 | 6,96E-14 | 1,2024E-18 |
| H1F0 | P07305 | ZCCHC8 | 28h | 1,66 | 2,41E-11 | 2,3408E-15 |
| OASL | Q15646 | ZCCHC8 | 0h | 1,67 | 6,59E-14 | 2,1574E-19 |
| OASL | Q15646 | ZCCHC8 | 28h | 1,72 | 5,71E-14 | 1,2329E-19 |
| ISG15 | P05161 | ZCCHC8 | 6h | 1,75 | 6,56E-13 | 2,4397E-15 |
| ISG15 | P05161 | ZCCHC8 | 28h | 1,87 | 5,8E-13 | 6,6631E-16 |
| ISG15 | P05161 | ZCCHC8 | 0h | 1,89 | 6,59E-14 | 5,8645E-16 |
| EPB41L3 | Q9Y2J2 | ZCCHC8 | 28h | 2,87 | 0,0265 | 2,1016E-08 |
| EPB41L3 | Q9Y2J2 | ZCCHC8 | 0h | 2,95 | 0,0127 | 1,3511E-08 |
| EPB41L3 | Q9Y2J2 | ZCCHC8 | 6h | 3,09 | 0,00684 | 6,2861E-09 |
| ACAT2 | Q9BWD1 | ZCCHC8 | 28h | 3,1 | 9,12E-10 | 9,9563E-15 |
| ACTL8 | Q9H568 | ZCCHC8 | 6h | 3,16 | 6,96E-14 | 6,9983E-30 |
| ACTL8 | Q9H568 | ZCCHC8 | 0h | 3,16 | 6,59E-14 | 6,8623E-30 |
| ACTL8 | Q9H568 | ZCCHC8 | 28h | 3,23 | 5,71E-14 | 4,5886E-30 |
| ACAT2 | Q9BWD1 | ZCCHC8 | 0h | 3,29 | 3,39E-12 | 3,2804E-15 |
| ACAT2 | Q9BWD1 | ZCCHC8 | 6h | 3,31 | 1,14E-12 | 2,9066E-15 |

| | | | | | | |
|---|---|---|---|---|---|---|
| ANXA1 | P04083 | ZCCHC8 | 28h | 4,17 | 5,71E-14 | 7,1906E-28 |
| ANXA1 | P04083 | ZCCHC8 | 6h | 4,25 | 6,96E-14 | 4,9758E-28 |
| ANXA1 | P04083 | ZCCHC8 | 0h | 4,25 | 6,59E-14 | 4,787E-28 |
| ALDH1B1 | P30837 | ZFC3H1 | 6h | -2,38 | 2,94E-14 | 3,2479E-17 |
| ALDH1B1 | P30837 | ZFC3H1 | 0h | -2,31 | 3,03E-14 | 5,5536E-17 |
| LRRC58 | Q96CX6 | ZFC3H1 | 0h | -2,02 | 3,03E-14 | 2,0415E-15 |
| LRRC58 | Q96CX6 | ZFC3H1 | 6h | -1,99 | 2,94E-14 | 2,8729E-15 |
| ALDH1B1 | P30837 | ZFC3H1 | 28h | -1,92 | 5,42E-14 | 2,1933E-15 |
| CRABP2 | P29373 | ZFC3H1 | 28h | -1,85 | 9,64E-13 | 5,3945E-15 |
| SERPINB5 | P36952 | ZFC3H1 | 28h | -1,71 | 2,77E-14 | 6,5047E-23 |
| ENO3 | P13929 | ZFC3H1 | 0h | -1,65 | 1,39E-10 | 9,9485E-14 |
| ENO3 | P13929 | ZFC3H1 | 6h | -1,64 | 9,08E-11 | 1,1467E-13 |
| ENO3 | P13929 | ZFC3H1 | 28h | -1,62 | 4,54E-09 | 1,4847E-13 |
| CRABP2 | P29373 | ZFC3H1 | 0h | -1,61 | 8,17E-11 | 8,0479E-14 |
| CTSZ | Q9UBR2 | ZFC3H1 | 28h | -1,6 | 0,0000096 | 1,3382E-11 |
| SERPINB5 | P36952 | ZFC3H1 | 0h | -1,53 | 3,03E-14 | 6,233E-22 |
| SERPINB5 | P36952 | ZFC3H1 | 6h | -1,51 | 2,94E-14 | 8,4566E-22 |
| SULT1A1 | P50225 | ZFC3H1 | 28h | -1,49 | 2,77E-14 | 4,3108E-19 |
| CRABP2 | P29373 | ZFC3H1 | 6h | -1,48 | 1,4E-09 | 3,6492E-13 |
| HPD | P32754 | ZFC3H1 | 28h | -1,47 | 2,77E-14 | 1,6042E-21 |
| LRRC58 | Q96CX6 | ZFC3H1 | 28h | -1,44 | 2,68E-07 | 1,2675E-12 |
| CTSC | P53634 | ZFC3H1 | 28h | -1,43 | 4,06E-09 | 1,4104E-13 |
| TRIM16 | O95361 | ZFC3H1 | 0h | -1,37 | 3,03E-14 | 1,4946E-17 |
| TRIM16 | O95361 | ZFC3H1 | 6h | -1,34 | 2,94E-14 | 2,2401E-17 |
| SULT1A1 | P50225 | ZFC3H1 | 0h | -1,34 | 3,03E-14 | 3,7459E-18 |
| ABCB1 | P08183 | ZFC3H1 | 28h | -1,33 | 0,00251 | 2,9509E-09 |
| SULT1A1 | P50225 | ZFC3H1 | 6h | -1,32 | 2,94E-14 | 4,8213E-18 |
| TRIM16 | O95361 | ZFC3H1 | 28h | -1,3 | 2,77E-14 | 4,3017E-17 |
| STAU1 | O95793 | ZFC3H1 | 28h | -1,29 | 2,26E-08 | 3,2454E-13 |
| CMBL | Q96DG6 | ZFC3H1 | 28h | -1,27 | 1,28E-11 | 1,3164E-14 |
| ZFC3H1 | O60293 | ZFC3H1 | 28h | -1,26 | 2,77E-14 | 3,9368E-18 |
| OPLAH | O14841 | ZFC3H1 | 0h | -1,25 | 3,03E-14 | 6,9623E-17 |
| CREG1 | O75629 | ZFC3H1 | 28h | -1,21 | 2,77E-14 | 2,805E-17 |
| PEG10 | Q86TG7 | ZFC3H1 | 28h | -1,21 | 4,08E-13 | 4,0655E-15 |
| OPLAH | O14841 | ZFC3H1 | 28h | -1,18 | 2,77E-14 | 2,3939E-16 |
| MTRR | Q9UBK8 | ZFC3H1 | 0h | -1,16 | 3,03E-14 | 2,5929E-18 |
| CTSC | P53634 | ZFC3H1 | 6h | -1,15 | 5,67E-07 | 7,9181E-12 |
| ABCB1 | P08183 | ZFC3H1 | 6h | -1,14 | 0,00646 | 3,8913E-08 |
| CTSZ | Q9UBR2 | ZFC3H1 | 6h | -1,14 | 0,00138 | 5,0697E-09 |
| OPLAH | O14841 | ZFC3H1 | 6h | -1,14 | 2,94E-14 | 4,5356E-16 |
| ZFC3H1 | O60293 | ZFC3H1 | 6h | -1,14 | 2,94E-14 | 2,661E-17 |
| LAMA1 | P25391 | ZFC3H1 | 0h | -1,13 | 0,0219 | 2,1348E-07 |
| MTRR | Q9UBK8 | ZFC3H1 | 6h | -1,12 | 2,94E-14 | 5,1806E-18 |
| MTARC1 | Q5VT66 | ZFC3H1 | 6h | -1,11 | 3,2E-13 | 1,5521E-14 |

| | | | | | | |
|---|---|---|---|---|---|---|
| ABCB1 | P08183 | ZFC3H1 | 0h | -1,11 | 0,0105 | 5,9025E-08 |
| FBXL6 | Q8N531 | ZFC3H1 | 28h | -1,1 | 0,00077 | 6,9382E-10 |
| MTARC1 | Q5VT66 | ZFC3H1 | 28h | -1,1 | 4,12E-11 | 2,0578E-14 |
| LAMA1 | P25391 | ZFC3H1 | 6h | -1,09 | 0,0237 | 3,4618E-07 |
| SLC1A3 | P43003 | ZFC3H1 | 28h | -1,09 | 0,00481 | 7,3779E-09 |
| CTSC | P53634 | ZFC3H1 | 0h | -1,08 | 0,0000056 | 2,5341E-11 |
| CMBL | Q96DG6 | ZFC3H1 | 6h | -1,07 | 1,13E-09 | 3,2785E-13 |
| CMBL | Q96DG6 | ZFC3H1 | 0h | -1,07 | 2,63E-09 | 3,4146E-13 |
| MTARC1 | Q5VT66 | ZFC3H1 | 0h | -1,07 | 8,64E-12 | 3,3249E-14 |
| NQO1 | P15559 | ZFC3H1 | 0h | -1,07 | 3,03E-14 | 1,2179E-15 |
| CTSZ | Q9UBR2 | ZFC3H1 | 0h | -1,06 | 0,0046 | 1,7361E-08 |
| HMGCS1 | Q01581 | ZFC3H1 | 0h | -1,06 | 3,03E-14 | 4,8815E-15 |
| HMOX1 | P09601 | ZFC3H1 | 28h | -1,06 | 2,77E-14 | 2,6627E-17 |
| THEM6 | Q8WUY1 | ZFC3H1 | 0h | -1,05 | 0,0000601 | 1,5497E-10 |
| MTRR | Q9UBK8 | ZFC3H1 | 28h | -1,05 | 2,77E-14 | 2,0017E-17 |
| HMGCS1 | Q01581 | ZFC3H1 | 6h | -1,04 | 2,94E-14 | 6,7646E-15 |
| SLC1A3 | P43003 | ZFC3H1 | 6h | -1,04 | 0,00338 | 1,5615E-08 |
| HPD | P32754 | ZFC3H1 | 6h | -1,03 | 2,94E-14 | 1,8E-18 |
| HPD | P32754 | ZFC3H1 | 0h | -1,03 | 3,03E-14 | 1,8126E-18 |
| NDRG1 | Q92597 | ZFC3H1 | 0h | -1,03 | 0,00000379 | 1,9298E-11 |
| RIMKLB | Q9ULI2 | ZFC3H1 | 0h | -1,03 | 0,000016 | 5,3697E-11 |
| LBR | Q14739 | ZFC3H1 | 6h | -1,02 | 1,51E-09 | 3,7896E-13 |
| NQO1 | P15559 | ZFC3H1 | 6h | -1,02 | 2,94E-14 | 3,0134E-15 |
| ERMP1 | Q7Z2K6 | ZFC3H1 | 0h | -1,02 | 3,03E-14 | 2,8977E-18 |
| RAI14 | Q9P0K7 | ZFC3H1 | 0h | -1,02 | 3,61E-10 | 1,4549E-13 |
| PIR | O00625 | ZFC3H1 | 28h | -1,02 | 2,11E-13 | 3,3259E-15 |
| SLC1A3 | P43003 | ZFC3H1 | 0h | -1,01 | 0,00595 | 2,4901E-08 |
| HMGCS1 | Q01581 | ZFC3H1 | 28h | -1,01 | 9,65E-12 | 1,1872E-14 |
| SH3BGRL3 | Q9H299 | ZFC3H1 | 6h | 1,01 | 0,0013 | 4,7297E-09 |
| JUNB | P17275 | ZFC3H1 | 28h | 1,01 | 0,00746 | 1,4247E-08 |
| SH3BGRL3 | Q9H299 | ZFC3H1 | 28h | 1,01 | 0,00354 | 4,7624E-09 |
| SH3BGRL3 | Q9H299 | ZFC3H1 | 0h | 1,02 | 0,00141 | 3,7419E-09 |
| MYPN | Q86TC9 | ZFC3H1 | 28h | 1,02 | 2,77E-14 | 3,3285E-16 |
| WARS | P23381 | ZFC3H1 | 6h | 1,03 | 2,94E-14 | 1,6916E-15 |
| GPX1 | P07203 | ZFC3H1 | 28h | 1,03 | 1,54E-08 | 2,6802E-13 |
| VAMP2 | P63027 | ZFC3H1 | 28h | 1,03 | 1,91E-08 | 2,9873E-13 |
| RCN3 | Q96D15 | ZFC3H1 | 6h | 1,04 | 2,13E-11 | 6,5623E-14 |
| EFHD1 | Q9BUP0 | ZFC3H1 | 0h | 1,04 | 0,00000124 | 9,4024E-12 |
| RCN3 | Q96D15 | ZFC3H1 | 0h | 1,04 | 6,71E-11 | 7,4176E-14 |
| NAB2 | Q15742 | ZFC3H1 | 28h | 1,04 | 1,03E-08 | 2,1983E-13 |
| NCBP2-AS2 | Q69YL0 | ZFC3H1 | 6h | 1,05 | 1,21E-09 | 3,3877E-13 |
| ECHDC3 | Q96DC8 | ZFC3H1 | 0h | 1,05 | 3,03E-14 | 4,9943E-17 |
| NES | P48681 | ZFC3H1 | 28h | 1,05 | 2,77E-14 | 6,987E-17 |
| NR4A1 | P22736 | ZFC3H1 | 28h | 1,05 | 0,0395 | 2,9614E-07 |

| | | | | | | |
|---|---|---|---|---|---|---|
| RCN3 | Q96D15 | ZFC3H1 | 28h | 1,05 | 6,15E-10 | 6,1467E-14 |
| ECHDC3 | Q96DC8 | ZFC3H1 | 6h | 1,06 | 2,94E-14 | 4,3842E-17 |
| WARS | P23381 | ZFC3H1 | 0h | 1,06 | 3,03E-14 | 1,0313E-15 |
| CYP1B1 | Q16678 | ZFC3H1 | 6h | 1,07 | 0,0169 | 1,8744E-07 |
| ISG15 | P05161 | ZFC3H1 | 6h | 1,07 | 0,00000383 | 2,6654E-11 |
| PTGES | O14684 | ZFC3H1 | 0h | 1,08 | 1,66E-09 | 2,7823E-13 |
| TFRC | P02786 | ZFC3H1 | 28h | 1,08 | 2,77E-14 | 1,8711E-15 |
| IFIT1 | P09914 | ZFC3H1 | 0h | 1,1 | 3,3E-08 | 1,155E-12 |
| PTGES | O14684 | ZFC3H1 | 6h | 1,15 | 4,67E-11 | 8,8221E-14 |
| EVPL | Q92817 | ZFC3H1 | 0h | 1,19 | 0,045 | 8,9241E-07 |
| ISG15 | P05161 | ZFC3H1 | 0h | 1,19 | 2,64E-07 | 3,5924E-12 |
| PTGES | O14684 | ZFC3H1 | 28h | 1,2 | 2,26E-10 | 4,008E-14 |
| UNC13D | Q70J99 | ZFC3H1 | 6h | 1,21 | 2,94E-14 | 1,4614E-19 |
| EVPL | Q92817 | ZFC3H1 | 6h | 1,22 | 0,0318 | 6,0077E-07 |
| SPRYD4 | Q8WW59 | ZFC3H1 | 28h | 1,22 | 1,07E-09 | 7,7953E-14 |
| UNC13D | Q70J99 | ZFC3H1 | 0h | 1,26 | 3,03E-14 | 7,4952E-20 |
| SLC12A7 | Q9Y666 | ZFC3H1 | 0h | 1,28 | 3,03E-14 | 1,0607E-17 |
| FOLR1 | P15328 | ZFC3H1 | 6h | 1,34 | 0,000238 | 7,493E-10 |
| ISG15 | P05161 | ZFC3H1 | 28h | 1,34 | 3,18E-08 | 3,8831E-13 |
| WARS | P23381 | ZFC3H1 | 28h | 1,34 | 2,77E-14 | 1,0082E-17 |
| SLC12A7 | Q9Y666 | ZFC3H1 | 6h | 1,35 | 2,94E-14 | 3,5974E-18 |
| OASL | Q15646 | ZFC3H1 | 28h | 1,35 | 2,77E-14 | 1,4971E-17 |
| FOLR1 | P15328 | ZFC3H1 | 0h | 1,36 | 0,000267 | 6,3124E-10 |
| DUSP9 | Q99956 | ZFC3H1 | 28h | 1,38 | 0,0265 | 1,3147E-07 |
| H1F0 | P07305 | ZFC3H1 | 28h | 1,39 | 8,53E-10 | 7,0728E-14 |
| FOLR1 | P15328 | ZFC3H1 | 28h | 1,41 | 0,000373 | 3,1338E-10 |
| DUSP9 | Q99956 | ZFC3H1 | 0h | 1,47 | 0,00894 | 4,5819E-08 |
| DUSP9 | Q99956 | ZFC3H1 | 6h | 1,48 | 0,00712 | 4,5122E-08 |
| OASL | Q15646 | ZFC3H1 | 6h | 1,52 | 2,94E-14 | 1,4595E-18 |
| SLC12A7 | Q9Y666 | ZFC3H1 | 28h | 1,52 | 2,77E-14 | 3,5269E-19 |
| OASL | Q15646 | ZFC3H1 | 0h | 1,66 | 3,03E-14 | 2,3392E-19 |
| PPL | O60437 | ZFC3H1 | 6h | 1,7 | 0,00349 | 1,6295E-08 |
| PPL | O60437 | ZFC3H1 | 0h | 1,7 | 0,00438 | 1,6271E-08 |
| SORBS2 | O94875 | ZFC3H1 | 6h | 1,73 | 2,94E-14 | 8,2249E-24 |
| SORBS2 | O94875 | ZFC3H1 | 0h | 1,75 | 3,03E-14 | 6,5646E-24 |
| SORBS2 | O94875 | ZFC3H1 | 28h | 1,84 | 2,77E-14 | 2,3637E-24 |
| NCBP2-AS2 | Q69YL0 | ZFC3H1 | 28h | 1,9 | 2,77E-14 | 3,8049E-18 |
| ACTL8 | Q9H568 | ZFC3H1 | 0h | 2,47 | 3,03E-14 | 9,8613E-28 |
| ACTL8 | Q9H568 | ZFC3H1 | 6h | 2,49 | 2,94E-14 | 8,4345E-28 |
| ACTL8 | Q9H568 | ZFC3H1 | 28h | 2,62 | 2,77E-14 | 3,0201E-28 |
| ANXA1 | P04083 | ZFC3H1 | 28h | 3,83 | 2,77E-14 | 4,0054E-27 |
| ANXA1 | P04083 | ZFC3H1 | 6h | 3,84 | 2,94E-14 | 3,8414E-27 |
| ANXA1 | P04083 | ZFC3H1 | 0h | 3,85 | 3,03E-14 | 3,6203E-27 |
| ACAT2 | Q9BWD1 | ZFC3H1 | 28h | 3,87 | 2,77E-14 | 1,3927E-16 |

| | | | | | | |
|---|---|---|---|---|---|---|
| ACAT2 | Q9BWD1 | ZFC3H1 | 0h | 3,95 | 3,03E-14 | 9,1706E-17 |
| ACAT2 | Q9BWD1 | ZFC3H1 | 6h | 3,98 | 2,94E-14 | 7,798E-17 |
