# Supplementary figures and images for: Rapid factor depletion highlights intricacies of nucleoplasmic RNA degradation
Source: Nucleic Acids Res. 2022 Jan 20;50(3):1583–600. doi: 10.1093/nar/gkac001 (PMC8860595; doi:10.1093/nar/gkac001)

Figure S1

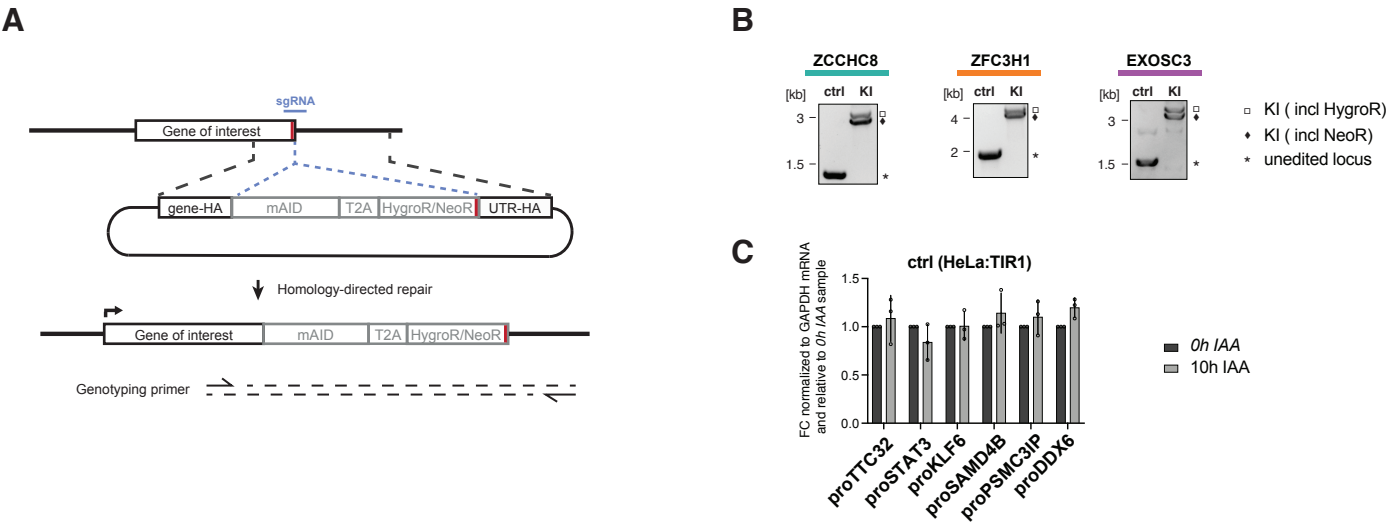

Supplement: gkac001_Supplemental_Files [file gkac001_supplemental_files.zip › figureS1.pdf]

Figure S2

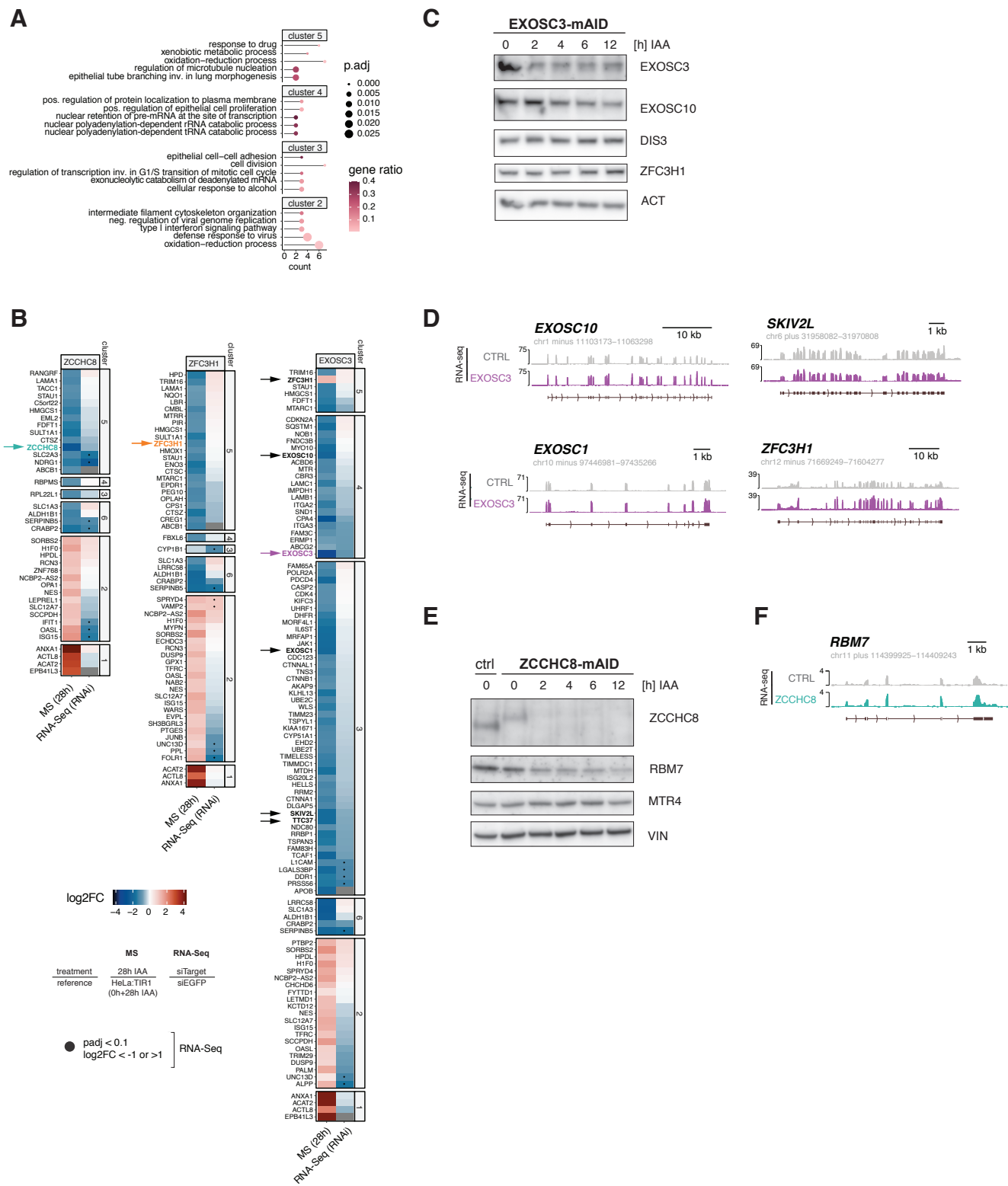

Supplement: gkac001_Supplemental_Files [file gkac001_supplemental_files.zip › figureS2.pdf]

Figure S3

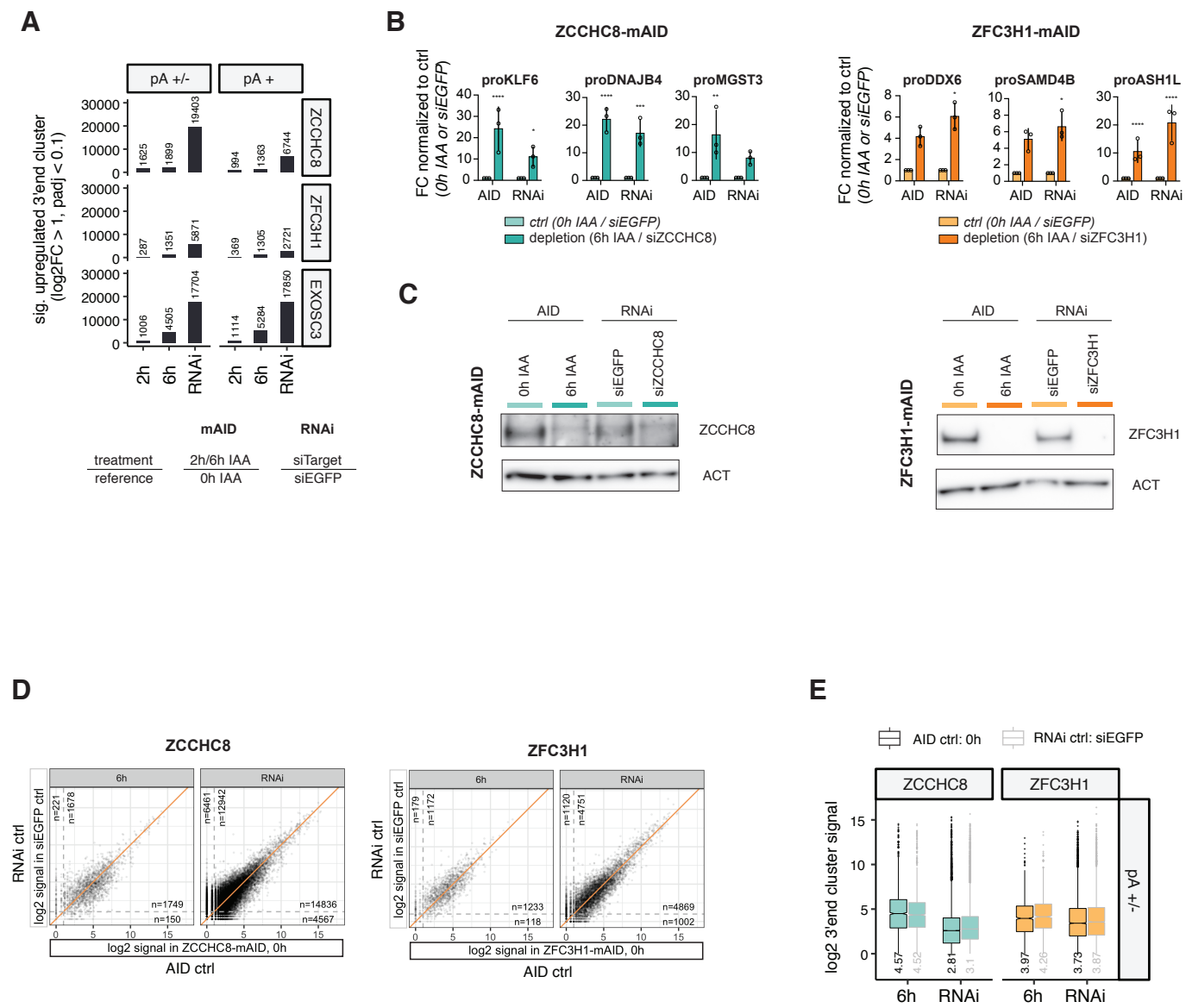

Supplement: gkac001_Supplemental_Files [file gkac001_supplemental_files.zip › figureS3.pdf]

Figure S4

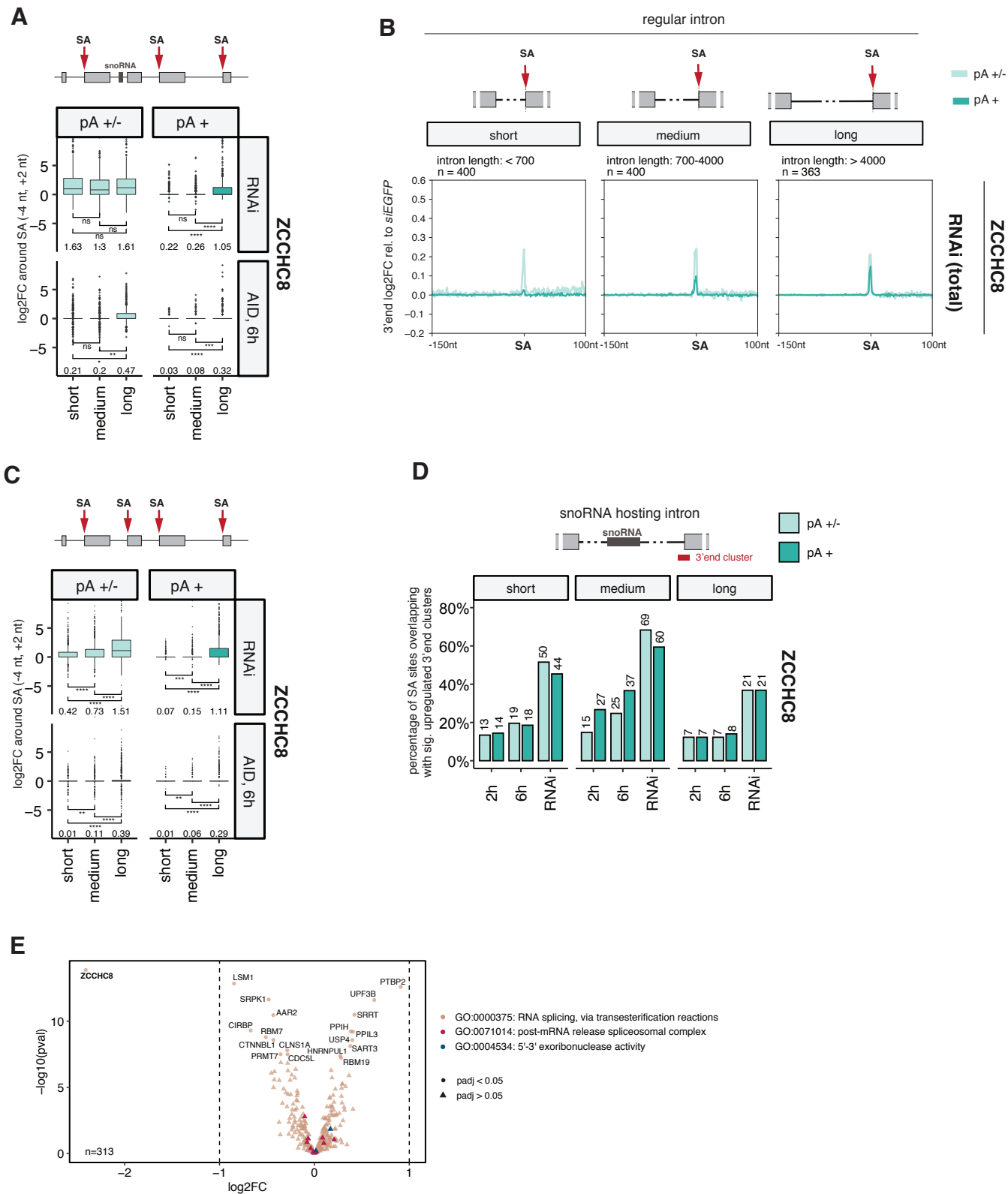

Supplement: gkac001_Supplemental_Files [file gkac001_supplemental_files.zip › figureS4.pdf]

Figure S5

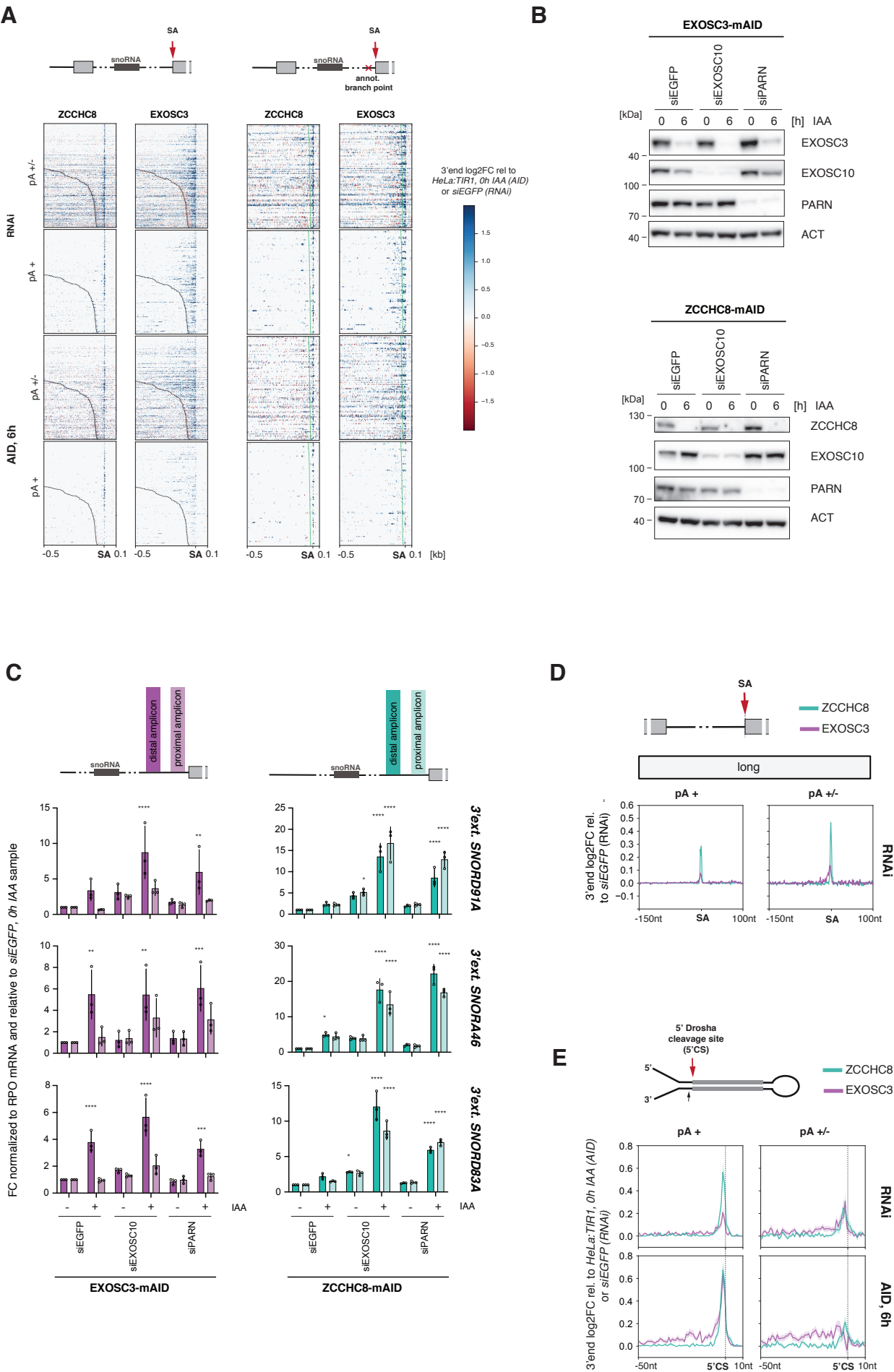

Supplement: gkac001_Supplemental_Files [file gkac001_supplemental_files.zip › figureS5.pdf]
